# Rapid, deep and precise profiling of the plasma proteome with multi-nanoparticle protein corona

**DOI:** 10.1038/s41467-020-17033-7

**Published:** 2020-07-22

**Authors:** John E. Blume, William C. Manning, Gregory Troiano, Daniel Hornburg, Michael Figa, Lyndal Hesterberg, Theodore L. Platt, Xiaoyan Zhao, Rea A. Cuaresma, Patrick A. Everley, Marwin Ko, Hope Liou, Max Mahoney, Shadi Ferdosi, Eltaher M. Elgierari, Craig Stolarczyk, Behzad Tangeysh, Hongwei Xia, Ryan Benz, Asim Siddiqui, Steven A. Carr, Philip Ma, Robert Langer, Vivek Farias, Omid C. Farokhzad

**Affiliations:** 1 Seer, Inc., Redwood City, CA 94065 USA; 2 Broad Institute of MIT and Harvard, Cambridge, MA 02142 USA; 3 David H. Koch Institute for Integrative Cancer Research, Massachusetts Institute of Technology, Cambridge, MA 02139 USA; 4 Sloan School and Operations Research Center, Massachusetts Institute of Technology, Cambridge, MA 02139 USA; 5 Center for Nanomedicine and Department of Anesthesiology, Brigham and Women’s Hospital, Harvard Medical School, Boston, MA 02115 USA

**Keywords:** Blood proteins, Mass spectrometry, Proteomics, Non-small-cell lung cancer, Nanoparticles

## Abstract

Large-scale, unbiased proteomics studies are constrained by the complexity of the plasma proteome. Here we report a highly parallel protein quantitation platform integrating nanoparticle (NP) protein coronas with liquid chromatography-mass spectrometry for efficient proteomic profiling. A protein corona is a protein layer adsorbed onto NPs upon contact with biofluids. Varying the physicochemical properties of engineered NPs translates to distinct protein corona patterns enabling differential and reproducible interrogation of biological samples, including deep sampling of the plasma proteome. Spike experiments confirm a linear signal response. The median coefficient of variation was 22%. We screened 43 NPs and selected a panel of 5, which detect more than 2,000 proteins from 141 plasma samples using a 96-well automated workflow in a pilot non-small cell lung cancer classification study. Our streamlined workflow combines depth of coverage and throughput with precise quantification based on unique interactions between proteins and NPs engineered for deep and scalable quantitative proteomic studies.

## Abstract (web summary)

Large-scale, unbiased proteomics studies of biological samples like plasma are constrained by the complexity of the proteome. Herein, the authors develop a highly parallel protein quantitation platform leveraging multi nanoparticle protein coronas for deep proteome sampling and biomarker discovery.

## Introduction

Broad-scale implementation of proteomic information in science and medicine has lagged behind genomics in large part because of the intricacies of protein molecules themselves and the lack of equivalent amplification mechanisms for low-abundance proteins. This has necessitated complex workflows that limit scalability making comprehensive studies of the plasma proteome exceptionally challenging. In spite of extensive efforts to interrogate the plasma proteome, relatively few new candidate biomarkers have been accepted as clinically useful^1–4^. Although the exact size of the plasma proteome is unknown, estimates range from >10,000 proteins to potentially covering all proteins^5^ with a concentration range exceeding 10 orders of magnitude, from albumin at 35–50 mg/mL to low-abundant proteins in the pg/mL range^6,7^. Combined with a lack of convenient molecular tools for protein analytical work (such as copy or amplification mechanisms), these features make comprehensive studies of the plasma proteome exceptionally challenging.

An extensive body of literature explores comprehensive, deep, and unbiased proteomic analysis of plasma and other biological samples by liquid chromatography-tandem mass spectrometry (LC-MS/MS)^3,5,8^. However, these studies often involve complex sample preparation workflows using immunodepletion of abundant proteins and chromatographic fractionation of samples upstream of LC-MS/MS analysis. More efficient techniques such as targeted analyte-specific (e.g., immunoassays) and untargeted LC-MS/MS proteomics strategies (without complex fractionation methods) have increased processing throughput, but lag behind the breadth and depth of proteomic coverage achieved with more work-intensive pipelines. Commercial targeted analyte-specific techniques can interrogate low- and high-abundance proteins and are amenable to multiplexing in the range of tens of proteins (e.g., Luminex and Meso Scale Diagnostics). Targeted MS has seen a dramatic expansion in utilization, either with simple fractionation methods (e.g. depletion of abundant proteins) or with anti-protein or anti-peptide immuno-enrichment workflows^9,10^. Nevertheless, even with these advances the number of targets remains only several hundred proteins^11,12^ and obviously requires prior knowledge of the targets to be measured.

Untargeted proteomics strategies with less work-intensive workflows enable enhanced throughput, but are generally limited to quantification of hundreds of predominantly higher-abundance proteins by LC-MS/MS^5,9^. Even with recent advances in parallel single-molecule protein sequencing^13^, the broad dynamic range of proteins in biological samples is still an obstacle to robust identification and quantification against a background of thousands of unique proteins, and even more protein variants^14,15^. While it is now possible to identify over 4500 proteins in plasma using advanced LC-MS/MS and data analytics^2,5,16^, these approaches generally rely on complex workflows including depletion, protein fractionation, peptide fractionation, and isobaric labelling coupled to LC-MS/MS, which is time-consuming (days to weeks), enforcing a trade-off between depth of protein coverage and sample throughput. These limitations not only hinder the discovery of new protein-based disease biomarkers, but constitute bottlenecks to faster adoption of proteogenomics and protein annotation of genomic variants^17^.

Increasing performance of proteomics pipelines in terms of throughput and depth can be achieved by at least two strategies: (1) employing advanced acquisition modes, like BoxCar^18^, scanning SWATH^19^ or state-of-the-art LC-MS setups such as ion mobility-enabled PASEF^20^ and sophisticated data processing pipelines that leverage additional information across and within samples^21–24^; and (2) improving the sample preparation, either by making low-abundant proteins and peptides more visible (increasing depth such as by fractionation and enrichment) or multiplexing samples to measure more samples in a shorter time (increasing throughput such as by isobaric labeling). These two strategies are often combined to increase performance. Despite advances in, and even when combined with sample preparation automation^25–27^, approaches that increase proteome coverage by sample preparation (strategy 2) usually make the workflow more complex and less scalable.

Nanoparticles (NP) that come into contact with a biological fluid such as plasma form a layer of proteins that coat the NPs at the nano-bio interface, which is referred to as a protein corona^28–30^. The effects of the protein corona on the biological fate of NPs in vitro and in vivo have recently been well explored^28–36^, and early studies focused on decreasing the binding of proteins and other macromolecules to the NP surface, commonly referred to biofouling, in an attempt to enhance utility for in vivo application^37–39^. Seminal systematic studies of the biophysics of protein corona formation then demonstrated the specificity of nano-bio interactions^31,34,35,40,41^. More recently we^36^ and others^41–46^ demonstrated that the composition and quantity of corona proteins depends largely on the physicochemical properties of the NP. Because altering these engineered properties reproducibly produces variation in the corona in terms of identity and/or quantity of proteins, it is now possible to systematically study the biomolecular information embedded within the protein corona of each unique NP.

Here, we describe a scalable and efficient protein identification and quantification platform that leverages the unique nano-bio interaction properties of multiple magnetic nanoparticles (NPs) with a protein corona strategy for highly parallel protein separation prior to MS. Our technology exploits magnetic NP-protein interactions and is therefore amenable to downstream sample processing such as multiplexing (e.g., isobaric labeling with tandem mass tag (TMT)) and any advanced MS acquisition strategy. Each NP interrogates hundreds of proteins across a broad dynamic range in an unbiased manner (e.g., not limited to a set of predetermined analytes, as in targeted or antibody-based strategies). We integrate multiple magnetic NPs in an automated Proteograph platform. Unlike other strategies that use single functionalized particles as a scaffold^47–50^, all NPs in the Proteograph platform are designed and engineered to synergistically, efficiently, and reproducibly sample complex proteomes based on the native physicochemical properties of proteins and unique nano-bio interactions. We characterize the assay linearity and precision possible with three NPs with distinct physicochemical properties demonstrating response linearity, signal reproducibility, and robustness. We also confirm the deeper sampling of the plasma proteome dynamic range by NP corona formation, enabling the capture and measurement of proteins spanning a wide dynamic range in a single LC-MS/MS run. Based on these results, we screen 43 NPs with distinct physicochemical properties to select a 10-particle panel optimized for plasma protein coverage. By comparison to published values^5^, we demonstrate that a panel of 10 NPs differentially samples the plasma proteome across more than seven orders of magnitude detecting 53 FDA-cleared protein biomarkers in a single pooled plasma. We test the utility for deep and rapid plasma proteome profiling in a pilot study distinguishing early non-small-cell lung cancer (NSCLC) subjects from age- and gender-matched healthy controls. We identify multi-protein classifiers including proteins known and unknown to play a role in NSCLC, supporting the NPs’ ability to identify new marker sets as the starting point for the eventual development of improved disease detection tests. The properties of our protein separation technology using multi-NP protein coronas present a scalable proteome sampling technology for deep unbiased proteomics to substitute for or complement existing sample preparation pipelines and integrate with any LC-MS/MS workflow.

## Results

### Engineering and characterizing NPs

**Fig. 1:**
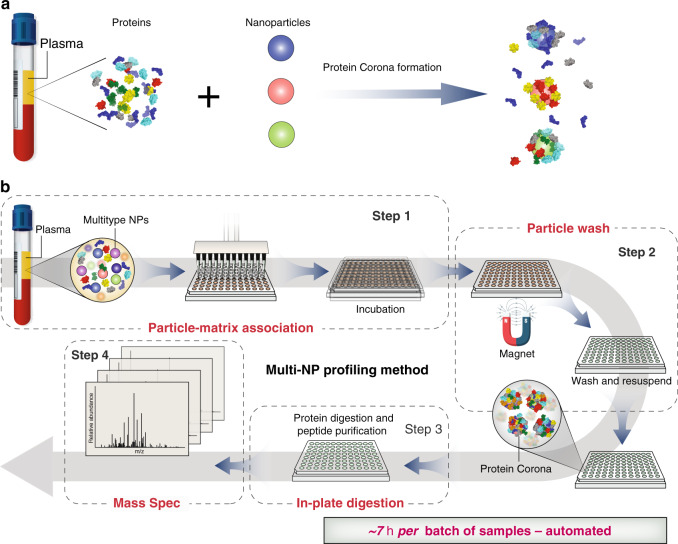
Schematic of workflow of the Proteograph. **a** Formation of NP protein corona. Different NP physicochemical properties (indicated by three different colors) led to the formation of different protein corona compositions on the NP surface. **b** Proteograph platform workflow based on multi-NP protein corona approach and mass spectrometry for plasma proteome analysis. The Proteograph workflow includes four steps: (1) NP-plasma incubation and protein corona formation; (2) NP protein corona purification by a magnet; (3) digestion of corona proteins; and (4) LC-MS/MS analysis. In this context, each plasma-NP well is a sample, for a total of 96 samples per plate.

**Fig. 2:**
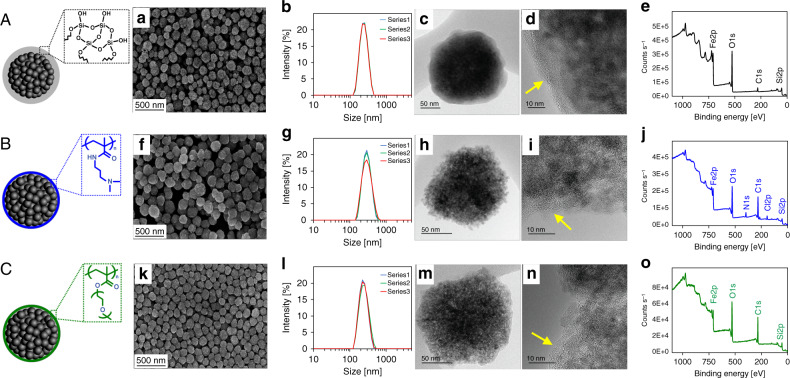
Characterization of the three SPIONs. A SP-003, B SP-007, and C SP-011, by **a**, **f**, **k** SEM, **b**, **g**, **l** DLS, **c**, **h**, **m** TEM, **d**, **i**, **n** HRTEM, and **e**, **j**, **o** XPS, respectively. DLS shows three replicates of each NP. Panels **d**, **i**, and **n** show the HRTEM pictures recorded at the surface of individual SP-003, SP-007, and SP-011 NPs, respectively, and the yellow arrow points to the region of **d** amorphous SiO_2_ coating and **i**, **n** amorphous SiO_2_/polymer coatings on the NP surface. Source data are provided as a Source Data file.

Various inorganic and organic NPs have been explored in fundamental studies of protein corona^29,34,36,40,46,51–53^. However, they may not be suitable for high-throughput translational proteomic analysis due to the necessity of repeated centrifugation or membrane filtration to separate the corona from free plasma proteins, and to wash away loosely attached proteins. In response, we developed superparamagnetic iron oxide NPs, or SPIONs (Figs. 1, 2a–c) for protein corona formation in an automatable assay, as the superparamagnetic core of the particle facilitates rapid magnetic separation from plasma (<30 sec) after corona formation (Supplementary Fig. 1), drastically reducing the time needed for extraction of NP protein corona for LC-MS/MS. Moreover, SPIONs can be robustly modified with different surface chemistries, which may facilitate the generation of distinct corona patterns for broader interrogation of the proteome (Supplementary Fig. 2).

Three SPIONs (SP-003, SP-007, and SP-011) with different surface functionalization were initially synthesized (Supplementary Table 1, Supplementary Fig. 3, Fig. 2) according to previously published methods^54–57^. SP-003 was coated with a thin layer of silica by a modified Stöber process using tetraethyl orthosilicate (TEOS). For the SPIONS coated with poly(dimethylaminopropyl methacrylamide) (PDMAPMA) (SP-007) and poly(ethylene glycol) (PEG) (SP-011), we first modified the iron oxide particle core with vinyl groups by a modified Stöber process using TEOS and 3-(trimethoxysilyl)propyl methacrylate. Next, the SPIONs were surface modified by free-radical polymerization with *N*-[3-(dimethylamino)propyl] methacrylamide (SP-007) or poly(ethylene glycol) methyl ether methacrylate (SP-011).

The three SPIONs were characterized in terms of size, morphology, and surface properties using techniques including scanning electron microscopy (SEM), dynamic light scattering (DLS), transmission electron microscopy (TEM), high-resolution TEM (HRTEM), and X-ray photoelectron spectroscopy (XPS) (Fig. 2). Our DLS measurements show that SP-003, SP-007, and SP-011 have average sizes/polydispersity indexes of, respectively, ~233 nm/0.05, ~283 nm/0.09, and ~238 nm/0.20. This is consistent with SEM showing that all three SPIONs are 200–300 nm with spherical and semi-spherical morphologies. Their surface charges of SP-003, SP-007, and SP-011 were evaluated by zeta potential (ζ) analysis, which shows the ζ values of, respectively, −36.9, +25.8, and −0.4 mV at pH 7.4 (Supplementary Table 1). This indicates negative, positive, and neutral surfaces, respectively, consistent with the coatings used (Fig. 2). Coating thickness was evaluated using HRTEM. For SP-003, an amorphous shell formed around the iron oxide core with a thickness >10 nm (Fig. 2d). For SP-007 and SP-011, a relatively thin (<10 nm) amorphous shell was formed (yellow arrows in Fig. 2i, n). In addition, XPS was performed for surface analysis, which, like HRTEM images, confirms the successful coating of the NPs with their respective functional groups.

The analytical results described above confirm that these three SPIONs constitute a diverse test set of NPs, which we further evaluated for protein detection coverage, precision, and linearity of response.

### Initial panel of three magnetic NPs for proteomic analysis

**Fig. 3:**
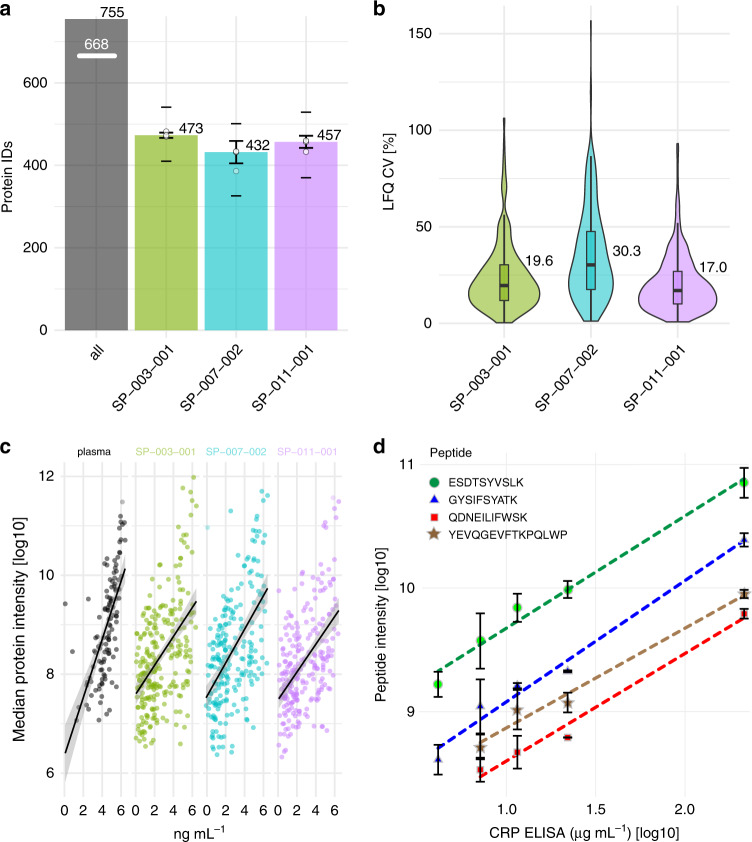
Proteomics characterization of the three initial SPIONs. **a** Protein groups from the NP corona of the three initial SPIONs, SP-003, SP-007, and SP-011 as determined by DDA LC-MSMS and MaxQuant (MaxLFQ, 1% protein and peptide FDR). All: represents proteins detected across all NPs. White line indicates the number of proteins detected with two or more peptides with at least one NP. For respective NPs median count and standard deviation across three assay replicates are shown as bar plots. Upper dashes depict number of proteins detected in any sample; lower dashes depict number of proteins detected in all three replicates. White circles show number of protein IDs for each assay replicate. **b** CV% for precision evaluation (MaxLFQ, filtering for three out of three valid values) of the NP protein corona-based Proteograph workflow. Inner boxplots report the 25% (lower hinge), 50%, and 75% quantiles (upper hinge). Whiskers indicate observations equal to or outside hinge ± 1.5 * interquartile range (IQR). Outliers (beyond 1.5 * IQR) are not plotted. Violin plots capture all data points. **c** Correlation of the maximum intensities of NP corona proteins vs. plasma proteins to the published concentration of the same proteins (median of assay triplicates). The black lines are linear regression models, and the grey shaded regions represent 95% confidence interval. **d** Linearity of response for measurement for CRP protein on the SP-007 NP in a spike-recovery experiment. Error bars denote standard deviations around the mean. All data were acquired in *n* = 3 independent assay replicates. Source data are provided as a Source Data file.

To evaluate the utility of our platform in proteomic analysis, we investigated the capacity of the three initial NPs to interrogate the complex proteome of blood plasma (Fig. 3, Supplementary Data 1). Each NP (100 µL) was first incubated with plasma for 1 h at 37 °C allowing for equilibrium of proteins that associate with NPs forming a stable protein corona, followed by magnet-based purification of NPs from unbound proteins (6 min per cycle × 3). The bound proteins were then digested, purified, and eluted. Notably, this highly parallel preparation workflow required only ~4–6 h in total for a batch of 96 corona preparations. The peptides from the NP-bound corona were analyzed in a 60-min LC-MS/MS run in data-dependent acquisition mode (DDA). Data were analyzed using MaxQuant for peptide identification and protein group assembly and MaxLFQ for quantification^58^.

Three NPs facilitated the quantification of >700 protein groups across nine samples (triplicate measurements of three NPs) and more than 500 protein groups with each nanoparticle type alone (Fig. 3a, Supplementary Table 2). For precision, we determined that detection of a protein in three out of three SPION coronas represents median CVs of 19.6%, 30.3%, and 17.0% (on average 22%) for SP-003, SP-007, and SP-011, respectively (Fig. 3b). The NP panel has sufficient precision to detect relatively small differences in fairly small studies. For example, in a study with just 25 samples and assuming 2000 measured analytes, we would have 85% power to detect differences of 50% in protein concentrations between groups with a Bonferroni-corrected alpha = 0.05/2000.

To explore the ability of NPs to interrogate plasma proteins present over a wide range of concentrations, we compared measured protein feature intensities from the protein coronas of the three NPs described above to published values^59^ (Fig. 3c). In parallel, we also directly measured peptides from a digested plasma sample without enrichment using SPIONs. The decreasing slopes for the fitted models for particle intensities indicate a reduction in the dynamic range of protein signal intensities as a function of abundance. This is consistent with previous observations^60,61^ that NPs can effectively reduce the measured dynamic range for abundances in the corona compared to the range in plasma by effectively normalizing protein abundance by binding affinity. Our multi-NP protein corona strategy thus facilitates the identification of a broad spectrum of plasma proteins, particularly those with low abundance, which pose challenges to rapid detection by conventional proteomic techniques.

To determine the linearity of our platform as a measurement tool and to support its utility in detecting true differences between groups of samples in biomarker discovery and validation studies, we first performed a spike-recovery study across four particles and three proteins comprising four polypeptides using Angiogenin, C-Reactive-Protein (CRP), Calprotectin (S100a8/9) (concentrations determined by ELISA: 3.3, 49, 8.9, and 8.9 ng/ml, respectively) and observed R^2^ between 0.90 and 1 (Supplementary Table 3, Supplementary Data 2). As exemplification, we present the results for SP-007 NP and C-reactive protein (CRP) in Fig. 3d. First, we used ELISA to determine the endogenous plasma level of CRP. Next, we spiked purified CRP (see Methods) to achieve testable multiples of the endogenous level. Post-spiking CRP levels were determined to be 4.11, 7.10, 11.5, 22.0, and 215.0 µg/mL corresponding to 1× (control), 2×, 5×, 10×, and 100× the endogenous level, respectively. We then plotted the quantities for the four indicated CRP peptides on the SP-007 NP versus the CRP concentrations as appropriate for comparing methods reporting different value types (Fig. 3d). Note that the MS1 feature intensity was undetectable for two of the CRP peptides in the unspiked plasma. The fitted lines are linear models using the given feature’s spike intensities.

Fitting a regression model to all four of the CRP tryptic peptides yielded a slope of 0.90 (95% CI 0.81–0.98) for the response of corona MS signal intensity versus ELISA plasma level, approaching perfect analytical performance. In contrast, a similar regression model fitted to 1308 other (nonspiked) MS features identified in at least four of the five plasma samples, for which signals from associated MS features should not vary across samples, had a slope of −0.086 (95% CI −0.1 to −0.068). These results indicate that the NPs’ linearity of response will likely prove useful in quantifying potential markers in comparative studies. Moreover, the response of the spiked-protein peptide features also suggests that with appropriate calibration, the NP protein corona method could be used to determine absolute, rather than relative, analyte levels.

Linearity of response was explored in greater depth with the addition of two other spiked proteins, Angiogenin and Calprotectin (S100a8/9), comprising three additional polypeptides and three additional NPs. The intensity data for these additional proteins and NPs were modeled against the measured ELISA values by linear regression, and a summary of the fits for the models is shown in Supplementary Table 3. The mean slope across all proteins and NPs is 1.06, indicating a linear response across the two orders of magnitude used in the spiked sample preparation (i.e., from 1× to 100× endogenous levels). The adjusted-R^2^ correlation for the intensities is also high (mean 0.95). These results confirm the linearity of response and indicate the ability of the NP platform to measure relative changes in peptide/protein levels across a broad range of concentrations with high precision.

To address the effect of background interference, we investigated the impact of varying lipid levels and extent of hemolysis: two common variables in plasma matrix composition. The lipid content of plasma changes not only with fasting state but also with age and state of health^62^. It is therefore important for every blood assay to be either insensitive to background matrix changes or to be able to control and correct for those introduced. We compared the number of identified proteins, the protein overlaps among conditions, and the intensity distributions measured from a pooled plasma sample spiked with low and high amounts of lipids, and subsequently treated with several NPs (Supplementary Figs. 4 and 5). Our data show that even high amounts of lipids do not affect the number or makeup of protein IDs or the intensity distributions compared to control samples with no lipid spikes. One tested NP (SP-356-001) shows a small reduction in protein IDs with high concentrations of spiked lipids when the sample is not centrifuged before measurement. This in fact highlights one of the advantages of using NPs: different surface properties could allow for the detection of biases comparing the coronas of particles for the same sample. We also observed good correlation in intensities across conditions, indicating the robustness of protein quantities.

Similarly, we investigated the effect of hemolysis using a human-derived red blood cell hemolysate spiked into a pooled plasma sample at low and high concentrations, as well as a control with no spike. As expected, cell debris introduced by hemolysis changes the protein count and content, as would be the case in any proteomics pipeline. However, proteins that overlap those detected in normal plasma are unaffected by the massively changing background introduced by hemolysis, as demonstrated by the correlation analysis (Supplementary Figs. 6 and 7).

### Optimized panel of 10 magnetic NPs

To further expand NP corona protein selection in a practicable format amenable to automation, we screened the coronas formed on 43 distinct SPIONs (Supplementary Data 3) in a similar fashion to the original three SPIONs. The goal was to select an optimized panel of 10 NPs that maximize the detection of proteins from a pooled plasma sample. The 43 candidate SPIONs were evaluated under six conditions (Methods), and the optimal conditions were used in a secondary analysis to select the best combination. The 43-SPION screen was conducted using pooled plasma from both healthy subjects and lung cancer patients (i.e., different from the pool used for the original three particles), to demonstrate platform validation across biological samples. In this analysis, a simpler criterion for protein detection was used for panel selection and optimization, i.e., a protein had to be represented by at least one peptide-spectral-match (PSM; 1% FDR) in each of three full-assay replicates to be counted as identified. The panel with the largest number of individual unique Uniprot identifiers was selected. This approach avoids any differential protein grouping effects possible across different combinations of evaluated NPs, since protein groups are based on the empirical data contained within any given analysis and might be confounded by the many diverse NP corona subsets.

**Fig. 4:**
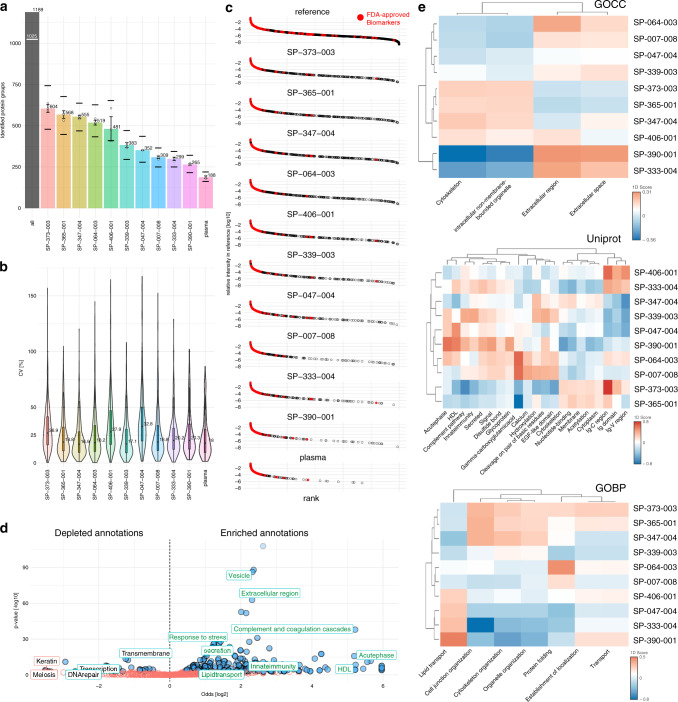
Optimized panel of 10 SPIONs in comparison to neat plasma. **a** Protein groups from the NP corona of 10 SPIONs, quantified by DDA LC-MS/MS (1% protein and peptide FDR). All: number of quantified protein groups across all NPs (excluding neat plasma). White line indicates the number of proteins detected with two or more peptides with at least one NP. For respective NPs median count and standard deviation across three assay replicates are shown as bar plots. Upper dashes depict number of proteins detected in any sample; lower dashes depict number of proteins detected in all three replicates. White circles show number of protein IDs for each assay replicate. **b** CV% distribution (precision) of the NP protein corona-based workflow for neat plasma and 10 SPIONs (filtering for three out of three valid values across assay replicates). Inner boxplots report the 25% (lower hinge), 50%, and 75% quantiles (upper hinge). Whiskers indicate observations equal to or outside hinge ± 1.5 * interquartile range (IQR). Outliers (beyond 1.5 * IQR) are not plotted. violin plots capture all data points. **c** Matching 10 SPIONs to a plasma protein database of MS intensities. Ranked intensities for the database proteins^5^ are shown in the top panel. Most intense protein is in the upper left corner of the panel; least intense is in the lower right corner. Intensities for proteins from neat plasma are shown in the bottom panel (plasma). Intensities for 10 SPIONs are shown in the remaining panels. Red dots indicated FDA-approved protein biomarkers^1^. **d** Volcano plot depicting annotation enrichment analysis (Fisher’s exact test) for functional pathways (GOCC,GOBP, KEGG, Uniprot Keywords, Pfam) of proteins detected in the optimized panel of 10 NPs in comparison to the database. Enriched = Log2 Odds > 0; depleted = Log2 Odds < 0. Blue circles indicated pathways with a Benjamini–Hochberg (B.H.) false discovery rate (FDR) < 1%. Green annotations indicate some enriched annotations enriched for NPs. Selected depleted annotatons are depicted in black. Keratin and Meiosis are depleted annotations with a B.H. FDR > 5%. **e** 1D annotation enrichment analysis comparing the protein intensity distribution (median intensity across assay triplicates, requiring three out of three quantifications) of each NP against the average of all. 1D scores are plotted as heat maps for annotations (minimal size 11) that are significantly enriched or depleted (2% B.H. FDR) for at least 1 NP. All data were acquired in *n* = 3 independent assay replicates. Source data are provided as a Source Data file.

The two-tiered screening approach described above yielded an optimized panel of 10 NPs with which we interrogated a common pooled plasma sample in three full-assay replicates (Fig. 4, Supplementary Fig. 8, Supplementary Data 4). We determined the median CVs for protein group quantification using MaxQuant (see Methods). The results ranged from 16.4 to 30.8% (Fig. 4b, Supplementary Table 4, 5), which is in the range of the precision determined for previous studies^4^.

Next we compared the precision of protein quantification to a published proteomics dataset. Given the large diversity in acquisition modes, quantification strategies, and protein inference pipelines, direct comparison of assay reproducibility is non-trivial. Geyer et al.^4^ describe a rapid LC-MS/MS proteomics approach with an abridged sample preparation protocol yielding an average of 284 protein groups per assay and 321 protein groups across all replicates. We found 88 identical protein groups between the 321 of Geyer et al. and our 1184 protein groups. Because protein groups can comprise multiple related proteins and assemble those proteins differently depending on the detected peptides, two mass spectrometry experiments can report partially overlapping protein groups. To allow as fair of a comparison as possible on the protein level, we compared the 88 protein groups that were composed of exactly the same Uniprot entries so there would be no ambiguity.

For these 88 common protein groups, we analyzed the data of Geyer et al.^4^ and found a median CV of 12.1% compared to a median CV across our NPs of 7.2%. We selected the NP that reports the best CV for each protein, as that is the one that would be selected for an assay. For a comparison from another perspective, Geyer also reports the number of protein groups with CVs < 20%, as this is a common cutoff for in vitro diagnostic assays. Our 10-NP panel detects 761 protein groups (with CV < 20%), which is 3.7 times greater than the number reported by Geyer^4^.

Next we investigated how the proteins detected with the 10-NP panel map to the abundance range of the plasma proteome (Fig. 4c). To this end, we mapped the proteins quantified with the 10-NP panel to the normalized intensities reported by Keshishian et al.^5^. In this study, more than 5000 protein groups were detected across 16 individual plasma samples in a complex workflow involving analysis of ~30 MS fractions per sample, taking a few weeks to complete^5^. Using the MS-derived plasma protein group intensities from that study, the coverage of each NPs was compared to this reference and to neat plasma (no depletion or enrichment). Proteins from neat plasma matching the database were skewed towards higher intensity (a proxy for abundance) in the full plasma protein database, whereas the protein constituents of the protein coronas from all 10 NPs extended nearly throughout the database’s entire dynamic range (Fig. 4c). Only 39 proteins in the database had intensities lower than the lowest protein group matched from a NP.

One key application of rapid, deep proteome analysis is the identification and quantification of protein biomarkers. While there are more than 100 FDA-cleared protein biomarkers^1^, the rate of the appearance of novel protein biomarkers per year is very low (less than 2 per year)^63^. In line with the observation made by Geyer et al.^3^, most biomarkers are in the high abundance range. Of the 90 mapped biomarkers, we identified between 33 and 43 within each of the NPs and in neat plasma (Fig. 4c, Supplementary Table 6, Supplementary Fig. 9).

While it is certainly important to compare the individual protein IDs, it is also of interest to determine which functional classes present in the reference plasma proteome are covered. To this end we mapped functional annotations (GOCC, GOBP, KEGG, Uniprot Keywords, Pfam) to Uniprot IDs and compared the enrichment and depletion of annotations in the panel of 10 NPs. Proteins covered with the 10 NPs panel showed significant enrichment for a variety of functional annotations including “secretion”, “innate immunity”, and “vesicles”. Underrepresented annotations include membrane- and DNA-associated annotations (Fig. 4d).

To further explore the capacity of individual NPs to interrogate different functional classes of proteins (i.e., extracellular region, membrane, or cytosol), we looked at NP-specific enriched annotations. For this analysis we employed a 1D annotation enrichment^64^ to compare protein coronas from individual NPs to the average profile of the entire 10-NP panel. Clustering based on 1D enrichment score (Fig. 4e) shows distinct and differential patterns of enrichment and depletion across the 10-NP panel. For example, GO Cellular Compartment annotations characterize protein location. In that category, NPs cluster into major branches (Cluster 1 with SP-373, SP-365, SP-347, and SP-406 versus Cluster 2/3 with SP-064, SP-007, SP-047, SP-339, SP-390, and SP-333). In contrast to Cluster 2 and 3, Cluster 1 shows depletion of proteins associated with the extracellular region and enrichment for intracellular proteins. Uniprot Keywords shows that some NPs specifically deplete for immune globulins (IgG) while showing enrichment for proteins annotated as secreted and involved in inflammation (e.g., SP-390, SP-339). Moreover, Uniprot Keywords and GO biological Process (GOBP) indicate that a subset of NPs, including SP-390 and SP-047, allow enrichment for lipid transport proteins, while other NPs like SP-007 could deplete proteins belonging to this functional class. In summary, annotation enrichments show that NP coronas can be categorized not only on the level of individual proteins but also based on functional groups of proteins. In principle, an experiment could take advantage of different subsets of particles focusing on specific protein group IDs or enriched annotations, whichever is more relevant to the question at hand. Moreover, the capacity to interrogate different functional classes of proteins (i.e., extracellular region, membrane, or cytosol) illustrates the capability of NP coronas to sample a wide dynamic range in complex proteomes.

### Large-scale application: non-small-cell lung cancer study

**Fig. 5:**
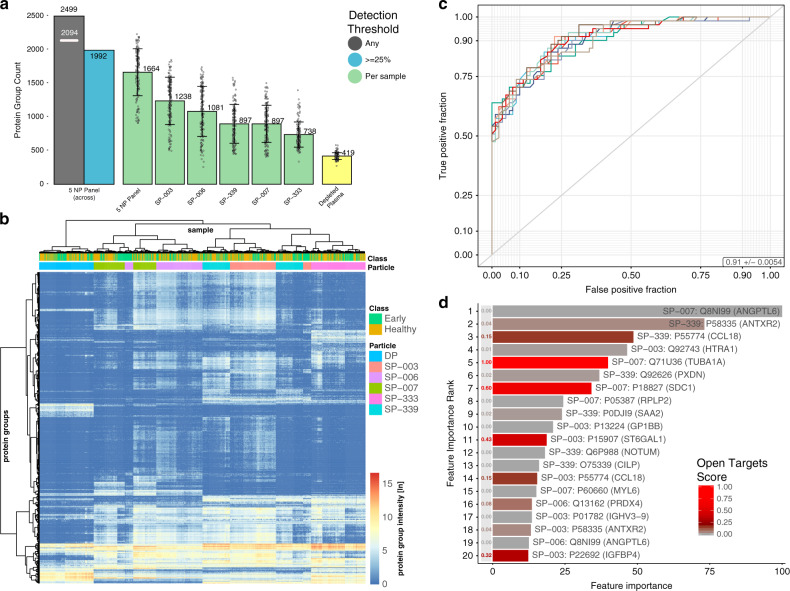
Classification of early NSCLC vs healthy using five NPs. **a** Protein group counts by NP and depleted plasma (filtered for 1% peptide and protein FDR). The green bars show the mean number of proteins in the cohort of 141 subjects found with the five NPs. The yellow bar shows the mean number of proteins in the cohort of 141 subjects for depleted plasma, the black bar shows the number of proteins across the five NP panel and all 141 subjects. The white line indicates the proteins that were detected with two peptides or more with one or more NP. The blue bar shows number of proteins across the five NP panel that were detected in at least 25% of all 141 subjects. Error bars depict standard deviation of identifications. White circles show number of protein IDs for each biological sample. **b** Heatmap showing the median normalized intensities (natural logarithm) of protein groups (rows) detected with five NPs (columns) or depleted plasma across 141 subjects (early NSCLC and healthy). Protein groups were filtered for 1% peptide and protein FDR and detection in at least 10% of the samples. Missing values were set to 0 (dark blue). Hierarchical clustering was performed in R using the ward.d2 method. **c** Receiver operating characteristic (ROC) curves quantifying the classification performance of healthy vs. early-stage NSCLC patients. Each colored curve represents one of the 10 repeats of the 10-fold cross validation where the performance was assessed on the hold-out test splits. The ROC average area under the curve (AUC) for across the 10 repeats is 0.91. **d** Top 20 most important features to classify healthy vs early NSCLC, with the color gradient showing the associated Open Targets Score for lung carcinoma targets. Source data are provided as a Source Data file.

To illustrate the performance of the Proteograph in a large human cohort, we performed a deep and rapid plasma proteome profiling of non-small-cell lung cancer (NSCLC) subjects and age- and gender-matched healthy and pulmonary comorbidity control subjects (Fig. 5; Supplementary Data 5–8, Supplementary Table 7). We used short a gradient (20 min gradient, 33 min sample-to-sample time) and a panel of five NPs selected from the original 10, optimized for maximum protein group coverage, in order to further reduce total experiment time. The total time required to complete these analyses was ~2 weeks. We evaluated precision using QC samples throughout the study, which showed that the Proteograph enables low CVs and a reproducible number of protein identifications even when processing more than 1500 assays measured across three mass spectrometers (five NPs and depleted plasma for each of the 141 subject samples).

To investigate the possibility of early NSCLC detection, we performed classification modeling on the sample set consisting of 80 healthy and 61 early-stage NSCLC subjects. On average, we identified 1664 proteins in these 141 subjects across five NPs (Fig. 5a). NPs composed distinct clusters for patterns of protein abundances (Fig. 5b). This unsupervised clustering analysis also showed a few subject specific differences but no clear pathology driven separation. We were particularly interested in how useful the additional proteins detected with NPs (beyond those detected in depleted plasma) are in stratifying healthy and NSCLC subjects, and removed the proteins detected in depleted plasma before building the classification models. The healthy vs early NSCLC classification achieved an average AUC of 0.91 (Fig. 5c) using a Random Forest model and 10 repeats of 10-fold cross validation. Random class permutation of the subjects achieved an average AUC of only 0.51, confirming the absence of overfitting in the classifier results. Examination of the top 20 classifier features (combination of particle and protein group), ranked by feature importance, highlights proteins both known and unknown to play a role in NSCLC as judged by Open Targets^65^ (OT) annotation (Fig. 5d). Among the most important features, we identified tubulin, which is the target of chemotherapeutic drugs including paclitaxel and its derivatives^66^.

In a recent study, Geyer et al. noted that the quality of clinical samples is often compromised by contamination with platelets and erythrocytes^67^. We checked which proteins of the most important classification features overlap with the deep platelet proteome published by Geyer et al.^67^. Only one of the top five features was detected in the platelet proteome (three other features with lower importance were also found in the remaining top 10). Notably, independent of the platelet index (see Methods) the Proteograph yields a considerably higher number of quantified proteins compared to depleted plasma (Supplementary Fig. 10, Supplementary Table 8).

## Discussion

Since early studies of biological protein association with the surface of NPs^30^, enormous strides have been made in understanding the protein corona, yielding numerous insights in nanomedicine and drug delivery^31–33^. It has increasingly been recognized that the protein corona determines the physiological responses to NPs (e.g., pharmacokinetics, biodistribution, cellular uptake, and therapeutic efficacy) and that NP-protein interactions are highly dependent on the NP’s physicochemical properties, exposure time, and protein source and concentration. More recently, ex vivo and in vitro interrogation of protein corona have been proposed for disease diagnosis and prediction^68–70^ and the LC-MS/MS proteomics analysis of protein corona formed on PEGylated liposomal doxorubicin (Caleyx™) after in vivo circulation has been shown to reveal low-abundance plasma proteins^46^.

Notwithstanding the above, little has been done to apply multiple NPs to the challenges of large proteomic biomarker studies that require broad protein coverage, deep dynamic range interrogation, and high sample throughput. The rationale for the current study is that small alterations to NP physicochemical properties can elicit dramatic but reproducible changes in protein corona^36,41–45^. We thus hypothesized that, compared to any single NP, multiple NPs with distinct engineered physicochemical properties offer expanded but partially overlapping proteomic sampling and more-comprehensive proteomics data.

We developed a highly parallel and automated protein separation technology platform (we refer to as Proteograph), which incorporates a panel of NPs selected from screening 46 engineered SPIONs with distinct physicochemical properties, into an ex vivo assay for protein corona formation and LC-MS/MS analysis, to achieve unbiased protein collection/detection. Using pooled plasma as a model complex biological sample, we validated our hypothesis that a larger NP panel identifies more proteins, particularly low-abundance proteins. In a panel of 10 NPs, we not only found distinct proteins but also protein pathways to associate with respective protein coronas. This suggests that addition of further distinct NPs should enable even broader and deeper proteome profiling. Thus, the platform can be tailored to profile the proteome at different levels by varying the number and type of NPs, analogous to different levels of coverage in gene sequencing. With the same NP panel, we detected 53 FDA-approved protein biomarkers. In agreement with previous observations^3^, most of these biomarkers were detected in the high-abundance range. Given the large number of low-abundance proteins NPs can detect, we predict that future studies will identify a number of novel biomarkers using a combination of NPs.

The multi-NP protein corona assay has also demonstrated several advantages in plasma proteome analysis. Unlike conventional deep proteomic techniques requiring depletion and fractionation workflows, our strategy is fast, scalable, and leverages physicochemical differences on the protein level without specifically targeting proteins. Notably, the multi-NP assay can be robustly automated and expanded by simply adding new NP variants, further increasing precision and breadth while speeding analysis in a 96-well plate format. Reproducibility and spike-recovery experiments also highlight the ability of our multi-NP protein corona platform to measure differences between samples, while reducing the concentration range of proteins in the enriched samples and facilitating detection of even low-abundance proteins, a key advantage of NP protein corona proteomic analysis. Since compressing the dynamic range affects measured abundance differences between different proteins within one sample, future studies could evaluate isotopically labeled protein spike-ins to calibrate measured quantities and derive absolute abundance information such concentrations or copy numbers.

In our NP-based classification feasibility study focusing on differentiation between samples from early-stage NSCLC patients and healthy controls, we demonstrated the utility of the platform to rapidly evaluate a large number of samples in a short period of time and identified novel combinations of known and unknown proteins as potential novel starting points for downstream NSCLC test development. In this study, more than 2000 proteins were quantified across 141 subjects in 2 weeks, a throughput enabled by the simplicity and robustness of the NP platform.

The performance of the healthy vs. early NSCLC (stages 1, 2, and 3) classifier was high (AUC 0.91), and we were able to identify proteins both known and unknown to play roles in NSCLC, supporting the value of proteins as an analyte class in developing better tests for early disease detection. Interestingly, among the most important features in the classification of healthy vs. early NSCLC, we identified tubulin, which—as a component of the cytoskeleton—is a usually intracellular protein detected in platelets^67^ but also a target for the chemotherapeutic paclitaxel and biomarker for neuronal tissue damage in cerebrospinal fluid^71^. Tissue damage and diseases like cancer could be associated with higher abundance of intracellular proteins that are otherwise correlated with contamination. New strategies to distinguish contamination markers from biological/ disease signatures are needed, in particular when interrogating complex physiological changes with highly sensitivity mass spectrometers. While this initial study provides a proof-of-concept for employing multiple NPs to identify protein biomarkers in a clinical cohort, these potential disease signatures have to be validated in follow-up studies.

The scalability and efficiency of our platform can fuel large proteomics studies, deepening our understanding of disease and biological mechanisms. It would be particularly interesting to integrate NPs into new mass spectrometry acquisition strategies such as BoxCar^18^, Scanning SWATH^19^, or ion mobility-enabled PASEF^20^. Another interesting possibility would be to use isobaric labeling (e.g., TMT) of peptides derived from our NP workflow to reduce MS run time by a factor of 10 or more. Despite the time advantage, isobaric labeling might be less suitable for some large-scale proteomic studies since it increases the costs of reagents and requires expensive MS3 capable instruments for the most accurate results^72^. Significant concurrent increase in the throughput of proteomic assay/analysis enabling larger size studies could help add proteomic data to large multiomic data sets to generate novel classifications and put genomic disease information that is still not well understood into functional context, such as single nucleotide polymorphism variants, changes in DNA methylation patterns, and splice variants. Moreover, protein-level information such as interactions or structural information are preserved on NP surface and can further elucidate functional context.

In addition, our NP technology could be extended and tailored to cerebrospinal fluid, cell lysates, and even tissue homogenates for rapid, accurate, and precise profiling of proteomes, facilitating discovery of new disease biomarkers. Furthermore, the multi-NP workflow addresses the dynamic range challenge at the intact protein level, and it is agnostic regarding the downstream protein identification and quantification strategy and can be integrated into low cost ELISA or emerging protein sequencing workflows. Ultimately, the broad utility of the functionalized multi-NPs workflow could be expanded into fields beyond proteomics, as NP surfaces can bind with any type of molecule. Possibilities include enrichment of nucleic acids for genomics, detection, and measurements of impurities in water sampling, and enhancing chemical sensing in environmental monitoring applications.

## Methods

### Materials

Iron (III) chloride hexahydrate ACS, sodium acetate (anhydrous ACS), ethylene glycol, ammonium hydroxide 28–30%, ammonium persulfate (APS) (≥98%, Pro-Pure, Proteomics Grade), ethanol (reagent alcohol ACS), and methanol (≥99.8% ACS) were purchased from VWR. *N,N*′-Methylenebisacrylamide (99%) was purchased from EMD Millipore. Trisodium citrate dihydrate (ACS reagent, ≥99.0%), tetraethyl orthosilicate (TEOS) (reagent grade, 98%), 3-(trimethoxysilyl)propyl methacrylate (MPS) (98%), and poly(ethylene glycol) methyl ether methacrylate (OEGMA, average Mn 500, contains 100 ppm MEHQ as inhibitor, 200 ppm BHT as inhibitor) were purchased from Sigma–Aldrich. 4,4′-Azobis(4-cyanovaleric acid) (ACVA, 98%, cont. ca 18% water) and divinylbenzene (DVB, 80%, mixture of isomers) were purchased from Alfa Aesar and purified by passing a short silica column to remove the inhibitor. N-(3-Dimethylaminopropyl)methacrylamide (DMAPMA) was purchased from TCI and also purified by passing a short silica column to remove the inhibitor. The ELISA kit to measure human C-reactive protein (CRP) was purchased from R&D Systems (Minneapolis, MN). Human CRP protein purified from human serum was from Sigma–Aldrich.

### Synthesis of NP SP-003, SP-007, and SP-011

The iron oxide core was synthesized following the published method via solvothermal reaction (Supplementary Fig. 3A)^54,55^. Typically, 26.4 g of iron (III) chloride hexahydrate was dissolved in 220 mL of ethylene glycol at 160 °C for ~10 min under mixing. Then 8.5 g of trisodium citrate dihydrate and 29.6 g sodium acetate anhydrous were added and fully dissolved by mixing for an additional 15 min at 160 °C. The solution was then sealed in a Teflon-lined stainless-steel autoclave (300 mL capacity) and heated to 200 °C for 12 h. After cooling to room temperature (RT), the black paramagnetic product was isolated by a magnet and washed with DI water 3–5 times. The final product was freeze-dried to a black powder for further use.

The silica-coated iron oxide NPs (SP-003) were prepared through a modified Stöber process as reported before (Supplementary Fig. 3B)^56,57^. Typically, 1 g of the SPIONs were homogeneously dispersed in a mixture of ethanol (400 mL), DI water (10 mL), and concentrated ammonia aqueous solution (10 mL, 28–30 wt%), followed by the addition of TEOS (2 mL). After stirring at 70 °C for 6 h, amorphous silica-coated SPIONs (denoted Fe_3_O_4_@SiO_2_) were washed three times with methanol, three times with water, and the final product was freeze-dried to a powder.

To prepare SP-007 (PDMAPMA-modified SPION) and SP-011 (PEG-modified SPION), vinyl group–functionalized SPIONs (denoted Fe_3_O_4_@MPS) were first prepared through a modified Stöber process as previously reported (Supplementary Fig. 3C)^41^. Briefly, 1 g of the SPIONs was homogeneously dispersed under the aid of vortexing (or sonication) in a mixture of ethanol (400 mL), DI water (10 mL), and concentrated ammonia aqueous solution (10 mL, 28–30 wt%), followed by the addition of TEOS (2 mL). After stirring at 70 °C for 6 h, 2 mL of 3-(trimethoxysilyl)propyl methacrylate was added into the reaction mixture and stirred at 70 °C overnight. Vinyl-functionalized SPIONs were obtained and washed three times with methanol, three times with water, and the final product freeze-dried to a powder. Next, for synthesis of poly(dimethylaminopropyl methacrylamide) (PDMAPMA)-coated SPIONs (denoted Fe_3_O_4_@PDMAPMA, SP-007 in Supplementary Fig. 3D), 100 mg of Fe_3_O_4_@MPS was homogeneously dispersed in 125 mL of DI water. After bubbling with N_2_ for 30 min, 2 g of *N*-[3-(dimethylamino)propyl] methacrylamide (DMAPMA) and 0.2 g of divinylbenzene (DVB) were added into the Fe_3_O_4_@MPS suspension under N_2_ protection. After the resulting mixture was heated to 75 °C, 40 mg of ammonium persulfate (APS) in 5 mL DI water was added and stirred at 75 °C overnight. After cooling, Fe_3_O_4_@PDMAPMA were isolated with a magnet and washed 3–5 times with water. The final product was freeze-dried to a dark brown powder. For synthesis of poly(ethylene glycol) (PEG)-coated SPIONs (denoted as Fe_3_O_4_@PEGOMA, SP-011 in Supplementary Fig. 3E), 100 mg of Fe_3_O_4_@MPS was homogeneously dispersed in 125 mL of DI water. After bubbling with N_2_ for 30 min, 2 g of poly(ethylene glycol) methyl ether methacrylate (OEGMA, average Mn 500) and 50 mg of *N,N*′-Methylenebisacrylamide (MBA) were added into the Fe_3_O_4_@MPS suspension under N_2_ protection. After the resulting mixture was heated to 75 °C, 50 mg of 4,4’-azobis(4-cyanovaleric acid) (ACVA) in 5 mL ethanol was added and stirred at 75 °C overnight. After cooling, Fe_3_O_4_@POEGMA were isolated with a magnet and washed 3–5 times with water. The final product was freeze-dried to a dark brown powder.

### Characterization of NP physicochemical properties

Dynamic light scattering (DLS) and zeta potential were measured on a Zetasizer Nano ZS (Malvern Instruments, Worcestershire, UK). NPs were suspended at 10 mg/mL in water with 10 min of bath sonication prior to testing. Samples were then diluted to ~0.02 wt% for both DLS and zeta potential measurements in respective buffers. DLS was performed in water at 25 °C in disposable polystyrene semi-micro cuvettes (VWR, Randor, PA, USA) with a 1 min temperature equilibration time and the average taken from three runs of 1 min, with a 633 nm laser in 173° backscatter mode. DLS results were analyzed using the cumulants method. Zeta potential was measured in 5% pH 7.4 PBS (Gibco, PN 10010-023, USA) in disposable folded capillary cells (Malvern Instruments, PN DTS1070) at 25 °C with a 1 min equilibration time. Three measurements were performed with automatic measurement duration with a minimum of 10 runs, a maximum of 100 runs, and a 1 min hold between measurements. The Smoluchowski model was used to determine the zeta potential from the electrophoretic mobility.

Scanning electron microscopy (SEM) was performed using a FEI Helios 600 Dual-Beam FIB-SEM. Aqueous dispersions of NPs were prepared to a concentration of 10 mg/mL from weighted NP powders re-dispersed in DI water by 10 min sonication. Then the samples were 4× diluted by methanol (Fisher) to make a dispersion in water/methanol that was directly used for electron microscopy. SEM substrates were prepared by drop-casting 6 µL of NP samples on the Si wafer from Ted Pella, and the droplet was completely dried in a vacuum desiccator for about 24 h prior to measurements.

A Titan 80–300 transmission electron microscope (TEM) with an accelerating voltage of 300 kV was used for both low- and high-resolution TEM measurements. The TEM grids were prepared by drop-casting 2 µL of the NP dispersion in a water-methanol mixture (25–75 v/v%) with a final concentration of 0.25 mg/mL and dried in a vacuum desiccator for about 24 h prior to TEM analysis. All measurements were performed on the lacey holey TEM grids from Ted Pella.

X-Ray Photoelectron Spectroscopy (XPS) was performed using a PHI VersaProbe and a Thermo Scientific ESCALAB 250e III. XPS analysis was performed on the NP fine powders kept sealed and stored under desiccation prior to measurement. Materials were mounted on carbon tape to achieve a uniform surface for analysis. A monochromatic Al K-alpha X-ray source (50 W and 15 kV) was used over a 200 µm^2^ scan area with a pass energy of 140 eV, and all binding energies were referenced to the C–C peak at 284.8 eV. Both survey scans and high-resolution scans were performed to assess in detail the elements of interest. The atomic concentration of each element was determined from integrated intensity of elemental photoemission features corrected by relative atomic sensitivity factors by averaging the results from two different locations on the sample. In some cases, four or more locations were averaged to assess uniformity.

### Protein corona preparation and proteomic analysis

Plasma and serum samples (BioIVT, Hicksville NY) were diluted 1:5 in a dilution buffer composed of TE buffer (10 mM Tris, 1 mM disodium EDTA, 150 mM KCl) with 0.05% CHAPS. NP powder was reconstituted by sonicating for 10 min in DI water followed by vortexing for 2–3 sec. To form the protein corona, 100 µL of NP suspension (SP-003, 5 mg/ml; SP-007, 2.5 mg/ml; SP-011, 10 mg/ml) was mixed with 100 µL of diluted biological samples in microtiter plates. The plates were sealed and incubated at 37 °C for 1 h with shaking at 300 rpm. After incubation, the plate was placed on top of a magnetic collection device for 5 min to draw down the NPs. Unbound proteins in supernatant were pipetted out. The protein corona was further washed with 200 µL of dilution buffer three times with magnetic separation.

For the 10-NP screen, the five additional assay conditions evaluated were identical to those described above, with one of the following exceptions. First, a low concentration of NPs was evaluated that was 50% the original concentration (ranging from 2.5–15 mg/ml for each NP, depending on expected peptide yield). For the second and third assay variations, both low and high NP concentrations were run using an undiluted, neat plasma rather than diluting the plasma in buffer. For the fourth and fifth assay variations, both low and high NP concentrations were run using a pH 5 citrate buffer for both dilution and rinse.

To digest the proteins bound onto NPs, a trypsin digestion kit (iST 96×, PreOmics, Germany) was used according to protocols provided. Briefly, 50 µL of Lyse buffer was added to each well and heated at 95 °C for 10 min with agitation. After cooling the plates to room temperature, trypsin digestion buffer was added, and the plate incubated at 37 °C for 3 h with shaking. The digestion process was stopped with a stop buffer. The supernatant was separated from the NPs by a magnetic collector and further cleaned up by a peptide cleanup cartridge included in the kit. The peptide was eluted with 75 µL of elution buffer twice and combined. Peptide concentration was measured by a quantitative colorimetric peptide assay kit from Thermo Fisher Scientific (Waltham, MA).

### NSCLC sample processing

As part of an ongoing, IRB-approved observational sample collection protocol, 24 sites were used to collect subject samples grouped into NSCLC (all stages, with 1, 2, and 3 referred to herein as early, and stage 4 defined as late), or healthy and pulmonary comorbid control arms. Subjects with pathology-confirmed NSCLC were enrolled post-diagnosis (typically achieved via a CT-guided fine-needle aspirant biopsy) but pretreatment. The protocol for obtaining blood samples from patients (Supplementary Note 1) was approved by the collections sites’ respective IRB’s (Supplementary Data 7), and all subjects gave written informed consent. Subjects were not necessarily fasted at the time of collection. Subjects for the pulmonary comorbidity control and healthy control groups were enrolled based on patient call-backs from participating study sites. In this context, healthy means the subjects did not have a current diagnosis of any form of cancer or any of the targeted pulmonary comorbidities including COPD, emphysema, etc. Sample types collected included EDTA plasma tubes, serum tubes, PAXgene RNA tubes, and Streck Blood Cell Collection tubes. For the purposes of this study, EDTA plasma was prepared as follows: After collection into the EDTA plasma tube per vendor instructions, the samples were centrifuged within 1 h of collection and the plasma fraction was aspirated and frozen within one hour of centrifugation prior to initial storage at −70 °C and subsequent shipment on dry ice. Study plasma samples were thawed at 4 °C, realiquoted, and refrozen once prior to NP processing. A randomly selected subcohort of 141 age- and gender-matched subjects from the healthy and early-stage NSCLC groups was selected for analysis from the collected samples with no significant differences between the groups based on Wilcoxon or Fisher tests, respectively. For NP analysis, the 141 plasma samples were randomized across sets of 96-well plates, one set for each NP. In addition to NP-plasma interrogation, a depleted plasma sample was prepared using the MARS-14 column (Agilent) per the manufacturer’s instructions. The NP-isolated peptides, as well as the peptides from equivalently digested depleted plasma, were evaluated by data-independent-acquisition mass spectrometry (DIA-MS) on Sciex Triple TOF 6600+ instruments coupled to an EKSPERT nano-LC 425 LC system running a 33 min sample-to-sample gradient. MS data acquisition took 2 weeks for all 141 samples.

### Data-dependent acquisition (DDA)

*LC-MS/MS*: Next, the peptide eluates were lyophilized and reconstituted in 0.1% TFA. A 2 µg aliquot from each sample was analyzed by nano-LC-MS/MS with either a Waters NanoAcquity HPLC system or a Thermo Scientific UltiMate 3000 RSLCnano system interfaced to an Orbitrap Fusion Lumos Tribrid Mass Spectrometer from Thermo Scientific. Peptides were loaded on a trapping column and eluted over a 75 µm analytic column at either 350 nL/min (NanoAcquity HPLC) or 250 nL/min (UltiMate 3000 RSLCnano system) using a gradient of 2–35% acetonitrile over 44 min, for a total time between injections of 64 (UltiMate 3000 RSLCnano system) or 66 min (NanoAcquity HPLC). The mass spectrometer was operated in data-dependent mode, with MS and MS/MS performed in the Orbitrap at 60,000 FWHM resolution and 15,000 FWHM resolution, respectively.

DDA Data Processing (all data excluding the NSCLC study): The MS data at the protein group level were acquired as follows. MS raw files were processed with MaxQuant/Andromeda (v. 1.6.7)^21,22^, searching MS/MS spectra against the UniProtKB human FASTA database (UP000005640, 74,349 forward entries; version from August 2019) employing standard settings. Enzyme digestion specificity was set to trypsin, allowing cleavage N-terminal to proline and up to 2 miscleavages. Minimum peptide length was set to seven amino acids and maximum peptide mass to 4600 Da. Methionine oxidation and protein N-terminus acetylation were configured as a variable modification, and carbamidomethylation of cysteines was set as a fixed modification. MaxQuant improves precursor ion mass accuracy by time-dependent recalibration algorithms and defines individual mass tolerances for each peptide. As initial maximum precursor mass tolerances, we allowed 20 ppm during the first search and 4.5 ppm in the main search. The MS/MS mass tolerance was set to 20 ppm. For analysis, we applied a false discovery rate (FDR) cutoff of 1% at both the peptide and protein level (protein groups are reported with their corresponding q-value). “Match between runs” was disabled. Identifications were quantified based on protein intensities (only proteins with q-value < 1%) requiring at least one razor peptide (Supplementary Data 3, 4). MaxLFQ^58^ normalized protein intensities (requiring at least one peptide ratio count) are reported in the raw output and were used only for the CV precision analysis. Proteins that could not be discriminated based on unique peptides were assembled in protein groups. Furthermore, proteins were filtered for a list of common contaminants included in MaxQuant. Proteins identified only by site modification were strictly excluded from analysis.

### Annotation-diversity analysis

To determine which annotations are predominantly enriched in the 10-NP panel (Fig. 4), we performed an annotation enrichment analysis using a Fisher’s exact test comparing proteins identified throughout the 10 NPs (requiring three out of three identifications across replicates) in a pooled plasma sample. Uniprot IDs (MaxQuant: Majority protein IDs) were matched to a list of 5304 published plasma proteins^5^ if any of the Uniprot IDs in the MaxQuant output matched the reported Uniprot ID. Next, annotations from five different spaces, GO Cellular Compartment (GOCC), GO Biological Process (GOBP), Uniprot Keywords, Protein families (Pfam), and Kyoto Encyclopedia of Genes and Genomes (KEGG), were matched to the protein groups based on Uniprot identifiers. Using Fisher’s exact test, we determined enriched annotations comparing the population of proteins identified by the 10 NPs within the reference database against the proteins that did not map into the 10-NP panel. Enrichment scores (Log2 Odds ratios) where calculated and plotted against the p-values (Fig. 4d). Annotations significantly enriched with a Benjamini–Hochberg FDR < 1% are indicated in blue. If log2 Odds were infinite, the maximum/ minimum log2 Odds where used for drawing.

We used continuous enrichment analysis (e.g., 1D annotation enrichment) to compare individual NPs at the annotation level, which has the advantage of using quantitative comparison, as a more powerful evaluation tool then requiring a binary input (e.g., presence/absence, threshold counting, etc.)^64^. We used this method to interrogate annotations enriched in the protein coronas by computing the 1D enrichment scores for each NP in the panel. In summary, log10-transformed MaxQuant intensities for each protein group in each sample were normalized by median subtraction. Protein groups that were not quantified in three out of three replicates used in the analysis on at least one NP were removed. A difference score was calculated for each protein group between the medians on one NP versus the average for that group across all of the other NPs. Annotations from five different spaces, GO Cellular Compartment (GOCC), GO Biological Process (GOBP), Uniprot Keywords, Protein families (Pfam), and Kyoto Encyclopedia of Genes and Genomes (KEGG), were matched to the protein groups based on the Uniprot identifiers reported in the MaxQuant output for each group as Majority Protein IDs. To match identifier format in the annotation reference, the isoform extensions were removed. The annotation references were retrieved from Uniprot on November 25, 2019 using the Perseus/MaxQuant framework^73^. The 1D annotation enrichment was calculated using R scripts adapted from the reported literature^64^. The results were filtered requiring (1) an annotation group size (i.e., number of protein groups with that annotation) greater than 10, and (2) a Benjamini–Hochberg-adjusted p-value (FDR) less than 2% for enrichment or depletion for at least one NP. The 1D enrichment score was visualized as a heatmap after hierarchical clustering as shown in Fig. 4e Gene Ontology Cellular Component (GOCC), B) Gene Ontology Biological Process (GOBP), C) Uniprot Keywords, D) Protein families (Pfam), E) Kyoto Encyclopedia of Genes and Genomes (KEGG). Hierarchical clustering is based on “complete linkage”.

### Data-independent acquisition (DIA), NSCLC study

*LC-MS/MS*: For DIA analyses using SWATH, peptides were reconstituted in a solution of 0.1% FA and 3% ACN spiked with 5fmol/uL PepCalMix from SCIEX (Framingham, MA). A constant mass of 5 ug of peptides per MS injection volume of 10 uL was targeted, but in some instances with lesser yield the maximum amount available was injected. Each sample was analyzed by an Eksigent nano-LC system coupled with a SCIEX Triple TOF 6600+ mass spectrometer equipped with OptiFlow source using a trap-and-elute method. First, the peptides were loaded on a trap column and then separated on an Eksigent ChromXP analytical column (150 mm × 15 cm, C18, 3 mm, 120 Å) at a flow rate of 5 uL/min using a gradient of 3–32% solvent B (0.1% FA, 100% ACN) over 20 min, resulting in a 33 min total run time. The mass spectrometer was operated in SWATH mode using 100 variable windows across the 400–1250 *m/z* range.

*Library generation for NSCLC study*: To build a peptide-spectral library, four plasma pools were created from the patients in the lung cancer. Each pool was analyzed by the Proteograph using the panel of 10 NPs. In addition, the four plasma pools were depleted using a MARS-14 column (Agilent, Santa Clara, CA) and the Agilent 1260 Infinity II HPLC system. The samples were analyzed in data-dependent mode on the UltiMate 3000 RSLCnano system coupled with Orbitrap Fusion Lumos using a gradient of 5–35% over 109 min, for a total run time of 125 min. The rest of the parameters were set as mentioned above.

To further expand the spectral library, a dataset from a separate experiment using a pooled plasma consisting of 157 healthy and lung cancer patients varying in age, gender, and disease stage was used in combination with the NSCLC-DDA data. In short, the pooled plasma was analyzed by the Proteograph assay using the panel of 10 NPs. Furthermore, the pooled plasma was depleted using the MARS-14 column and fractionated into nine concatenated fractions using a high-pH fractionation method (XBridge BEH C18 column, Waters). All samples were prepared in three replicates and analyzed in data-dependent mode using the same parameters as NSCLC-DDA analysis.

*Plasma depletion*: All depleted plasma samples were prepared using an Agilent 1260 Infinity II Bioinert HPLC system consisting of autosampler, pumps, column compartment, UV detector, and fraction collector. Plasma depletion was conducted by first diluting 25 μL of plasma to a final volume of 100 μL using Agilent Buffer A plasma depletion mobile-phase. Each diluted sample was filtered through an Agilent 0.22 μm cellulose acetate spin filter to remove any particulates and transferred to a 96-well plate. The plate was then placed in an autosampler and held at 4 °C for the entirety of the assay. Eighty microliters of the diluted plasma was then injected onto an Agilent 4.6 × 50 mm Human 14 Multiple Affinity Removal System (MARS-14) depletion column housed in the column compartment at a constant temperature of 20 °C. Mobile-phase conditions used during protein depletion consisted of 100% Buffer A mobile-phase flowing at a rate of 0.125 mL/min. Proteins eluting from the column were detected using the Agilent UV absorbance detector operated at 280 nm with a bandwidth of 4 nm. The early eluding peak for each injection, representing the depleted plasma proteins, was collected using a refrigerated fraction collector with peak-intensity based triggering (i.e., 200 mAu threshold with a maximum peak width of 3 min). After peak collection, the fractions were held at 4 °C for the duration of the analysis. The sample volume was then reduced to approximately 20 μL using an Amicon Centrifugal Concentrator (Amicon Ultra-0.5 mL, 3k MWCO) with a centrifuge operating at 4 °C and 14,000 × *g*. Five microliters of each depleted sample was then reduced, alkylated, digested, desalted, and analyzed according to the sample preparation and MS analysis protocols described. During each sample depletion cycle, the MARS-14 column was regenerated with the Agilent Buffer B mobile-phase for ~4 ½ min at a flow rate of 1 mL/min and equilibrated back to the original protein capture condition by flowing Buffer A at 1 mL/min for ~9 min.

*Peptide fractionation*: A total of 100 μl of reconstituted peptides was loaded to a Waters XBridge column (2.1 × 250 mm, BEH C18, 3.5 mm, 300 Å) using the Agilent 1260 Infinity II HPLC system. The peptides were separated at the flow rate of 350 mL/min using a gradient of 3–30% in 30 min, with a total run time of 47 min, and the fractions were collected every 1.5 min. The fractions were then dried using a speed vac. Finally, the dried peptides were reconstituted in a solution of 0.1% FA and 3% ACN and concatenated to 9 fractions.

*Data analysis for library generation*: To generate a spectral library, all the DDA data were first searched against human Uniprot database using the Pulsar search engine in Spectronaut (Biognosys, Switzerland). Then the library was generated using Spectronaut with 1% FDR cutoff at peptide and protein level.

*DIA raw data processing*: The SWATH data were processed on Spectronaut. The default settings (version 13.8.190930.43655) were used for the analysis with the Q-value cutoff at precursor and protein level set to 0.01 (Supplementary Data 5).

For classification analysis (NSCLC study), primary MS data were prepared as follows. Statistical analysis was performed using the R platform as described above including the core ‘tidyverse‘ packages, the ‘caret‘ classification framework and the ‘ranger‘ random forest model package. Missing values for a given protein group within a subject were median imputed. No other normalization was applied to the data prior to classification. In order to construct between-group classifier models, log-transformed protein group data were evaluated in ten rounds of 10-fold cross validation. All protein group features were used for classification and the relative importance of those features in the cross-validations was reported. In order to detect possible overfitting, ten iterations of the cross-validation procedure were performed after randomization of the subjects’ class assignments. Initial classification results highlighted a significant signal from both the depleted plasma and NP panel data from proteins typically associated with stress and acute-phase response, likely a result of the sample acquisition strategy (e.g., post biopsy, diagnosis-aware). To eliminate this possibly confounding signal, all protein group data from the NP-derived dataset that was derived from any protein also observed in depleted plasma was removed from subsequent analysis.

### Platelet Index (PI)

Protein groups identified in a sample by particle were matched to the platelet signature protein list from Geyer et al.^67^, and the sample platelet index (PI) was calculated as the median of the ln intensity of the signature proteins divided by the median of the ln intensity of the non-signature proteins. In order to summarize an overall PI for the sample from all particles and depleted plasma, the PIs for each particle were scaled and centered (default scale() R function) and the average was taken across the six values (five NPs and DP).

### Spike recovery

Baseline concentration of CRP in a pooled healthy plasma sample was measured with the ELISA kit as described above (Materials) according to the manufacturer-suggested protocols. A stock solution and appropriate dilutions of CRP were prepared and spiked into the identical pooled plasma samples to make final concentrations 2×, 5×, 10×, and 100× baseline endogenous concentrations. The volume of additions to the pooled plasma was 10% of the total sample volume. A spike control was made by adding the same volume of buffer to the pooled plasma sample. Concentrations of spiked samples were measured again by ELISA to confirm the CRP levels in each spiking level. The samples were used to evaluate Proteograph NP corona measurement linearity as described in the Results above.

### Background robustness test

Interference substances were obtained from Sun Diagnostics. Lipids: Triglyceride-rich lipoproteins derived from human. Hemolysate: Red blood cell hemolysate derived from human. A pooled plasma was spiked at different concentrations Lipid: High (1000 mg/dL), Low (100 mg/dL), and Control (buffer only). Hemolysate: High (1000 mg/dL), Low(100 mg/dL), and Control (buffer only).

### Statistics and reproducibility

Statistical analysis and visualization were performed using R (v3.5.2) with appropriate packages^74^. Experiments were conducted in assay replicates (*n* = 3) unless noted differently. NSCLC data were acquired for biological replicates (see above). Mass spectrometry raw data and functional protein annotation references are available through PRIDE^75^ and Perseus^76^, respectively.

### Reporting summary

Further information on research design is available in the Nature Research Reporting Summary linked to this article.

## Supplementary information

Supplementary Information

Description of Additional Supplementary Files

Supplementary Data 1

Supplementary Data 2

Supplementary Data 3

Supplementary Data 4

Supplementary Data 5

Supplementary Data 6

Supplementary Data 7

Supplementary Data 8

Reporting Summary

## Data Availability

NSCLC study clinical and participant information are provided in Supplementary Data 7 and 8. The mass spectrometry proteomics data (Figs. 3, 4, 5 and associated analyses) have been deposited to the ProteomeXchange Consortium (http://proteomecentral.proteomexchange.org) via the PRIDE partner repository^75^ with the dataset PXD017052. Annotations used for annotation enrichment analysis (Figs. 4 and 5) are available as part of the Perseus^76^ framework. The Uniprot Fasta is available on https://www.uniprot.org/ (retrieved 2019-08-29). All other data are available from the corresponding authors on reasonable request. Source data are provided with this paper.

## Source data

Source Data
