# Supplementary material for: Rapid, deep and precise profiling of the plasma proteome with multi-nanoparticle protein corona
Source: Nat Commun. 2020 Jul 22;11:3662. doi: 10.1038/s41467-020-17033-7 (PMC7376165; doi:10.1038/s41467-020-17033-7)
Supplement: Supplementary file 1 — Supplementary Information [file 41467_2020_17033_MOESM1_ESM.pdf]

Supplementary Information for

Rapid, Deep and Precise Profiling of the Plasma Proteome with Multi-Nanoparticle Protein Corona

Blume J., Manning W.C. , Troiano G., Hornburg D. et al.

A **Proteograph Platform**

- parallel processing of 80 plasma-nanoparticle assays
- ~ 7h fully automated workflow for entire sample prep
- > 1,500 proteins quantified in plasma per sample in 2.5 h (MS time)

B

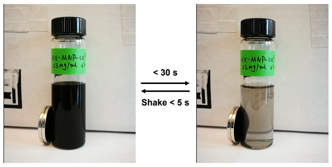

C

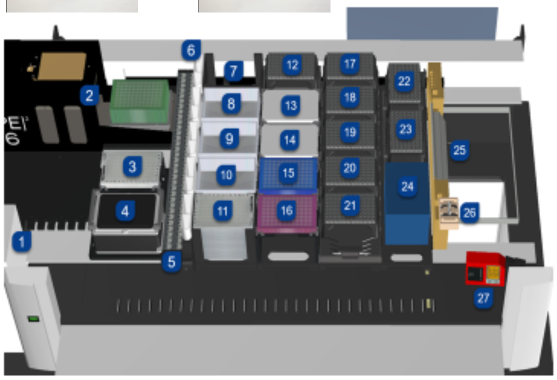

Number	Description	Number	Description
1	CO-RE 96 Probe Head	15	Plate Carrier Position
2	$[MPE]^2$	16	Plate Carrier Position
3	Magnet Position	17	NTR Stack Module for MPH
4	Hamilton Heater Shaker (HHS)	18	NTR Stack Module for MPH
5	Magnet Position	19	NTR Stack Module for MPH
6	Nanoparticle & Plasma Sample Tubes	20	NTR Stack Module for MPH
7	Plate Stack Module	21	NTR Stack Module for MPH
8	TE Buffer Reservoir	22	NTR Stack Module for Channels
9	Wetting Reagent (100% MeOH) Reservoir	23	NTR Stack Module for Channels
10	Condition Reagent (H20) Reservoir	24	Inheco CPAC
11	Plate Stack Module	25	Tip Waste
12	NTR Module	26	CO-RE Paddles
13	Lid Park Position	27	Autoload
14	Lid Park Position	28	-

©2019 Hamilton Company 5

Supplementary Figure 1 Automation workflow

A) Proteograph rapid and fully automated samples processing for deep plasma proteome analysis. 5 NPs yield more that 1500 protein groups on average when analyzed in 30 min DIA-MS runs each. B) Rapid separation of SPIONs – superparamagnetic iron oxide nanoparticles (NPs). C) customized STARlet Deck layout for sample processing.

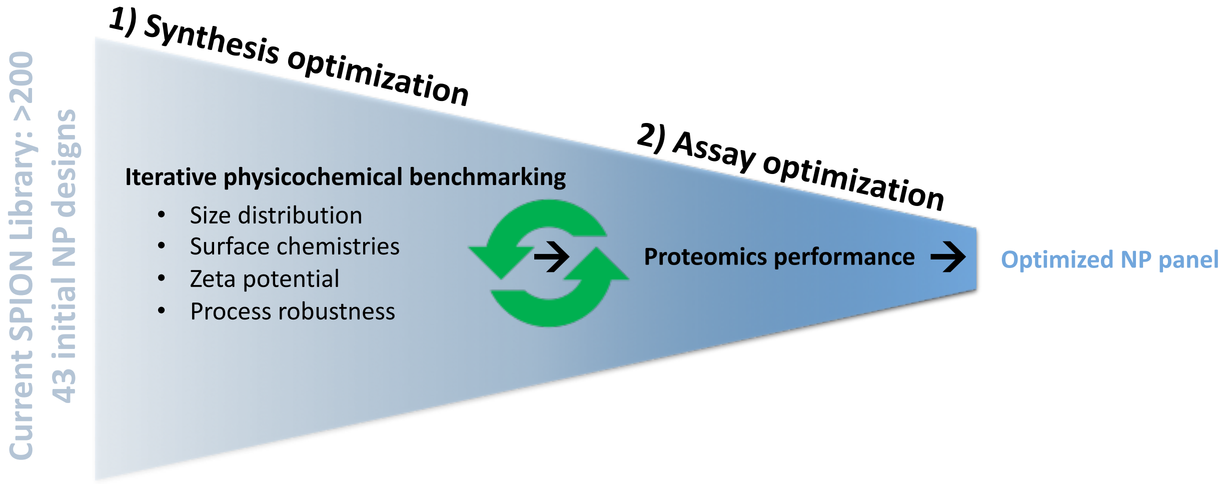

Supplementary Figure 2. SPION library synthesis approach and optimization

We design and develop SPION panels through a combinatorial optimization process. First, we create a library of SPIONs in our nanotechnology formulations lab through a combination of rational design and empiricism. We currently have over 200 SPIONs in our particle library. A fraction of these are eliminated or screened out based on physicochemical properties alone, such as poor handling properties or lack of reproducible synthesis. We selected 46 SPIONS and gathered proteomic data using mass spectrometry from the protein corona formed on these particles. Then we select a subset of 10 particle panel based on their ability to sample the proteome broadly and deeply. The scientific rationale and efforts behind the engineering and selection of 10 particle panel is now described in the SI section of the revised manuscript.

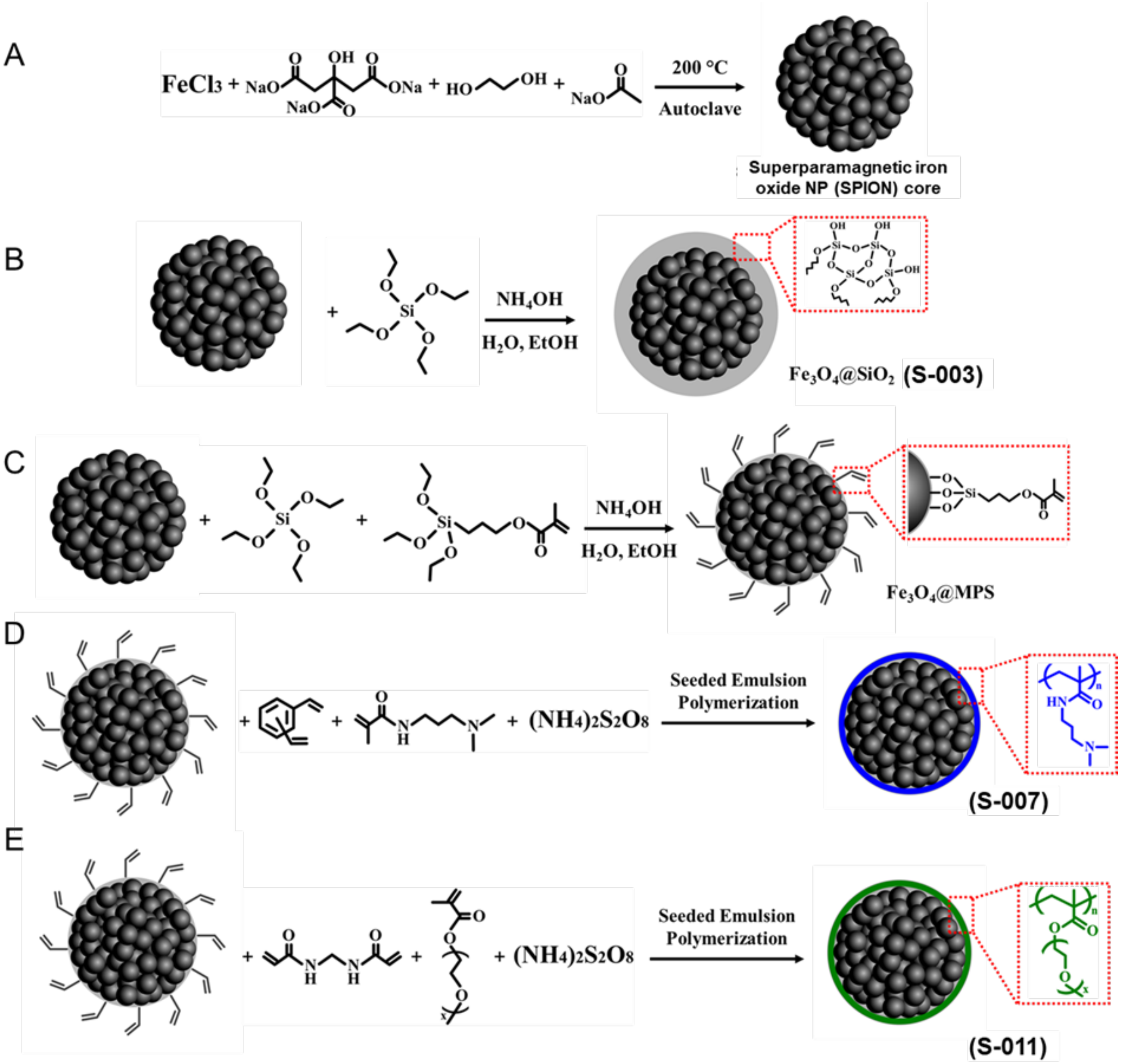

Supplementary Figure 3 Schematic for synthesis

A) SPION core, B) silica-coated SPION (SP-003), C) vinyl group–functionalized SIONP, D) poly(N-(3-(dimethylamino)propyl) methacrylamide) (PDMAPMA)-coated SPION (SP-007), and E) poly(oligo(ethylene glycol) methyl ether methacrylate) (POEGMA)-coated SPION (SP-011).

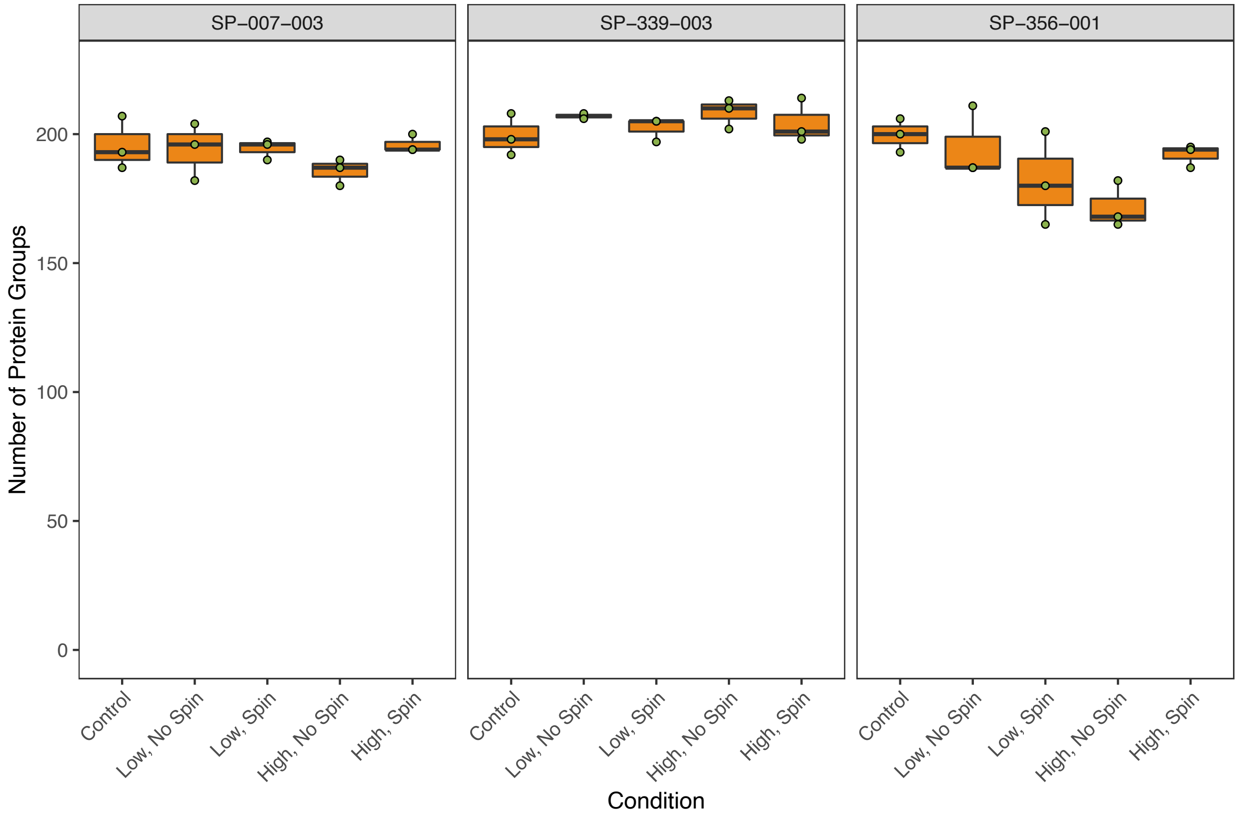

Supplementary Figure 4 Lipid interference identification

The lipid interference runs tested 2 levels of spiked lipids (low and high), as well as a control with no spiked lipids. Because the samples appeared cloudy with the addition of lipids, lipid-spiked samples were either spun or not spun prior to the assay (5 different conditions total). Each spike-level/spin condition was run in triplicate and tested against 3 nanoparticles. The number of detected protein groups across the conditions and particles is shown in the figure. SP-356-001 showed more variability in the counts across the conditions compared to the other particles. The counts were fairly consistent with CV's (of the counts) between ~3.0% - 7.6% within the particles. Boxplots report the 25% (lower hinge), 50%, and 75% quantiles (upper hinge). Whiskers indicate observations equal to or outside hinge + / – 1.5 * interquartile range (IQR). All data was acquired in n = 3 independent assay replicates. Source data are provided as a Source Data file.

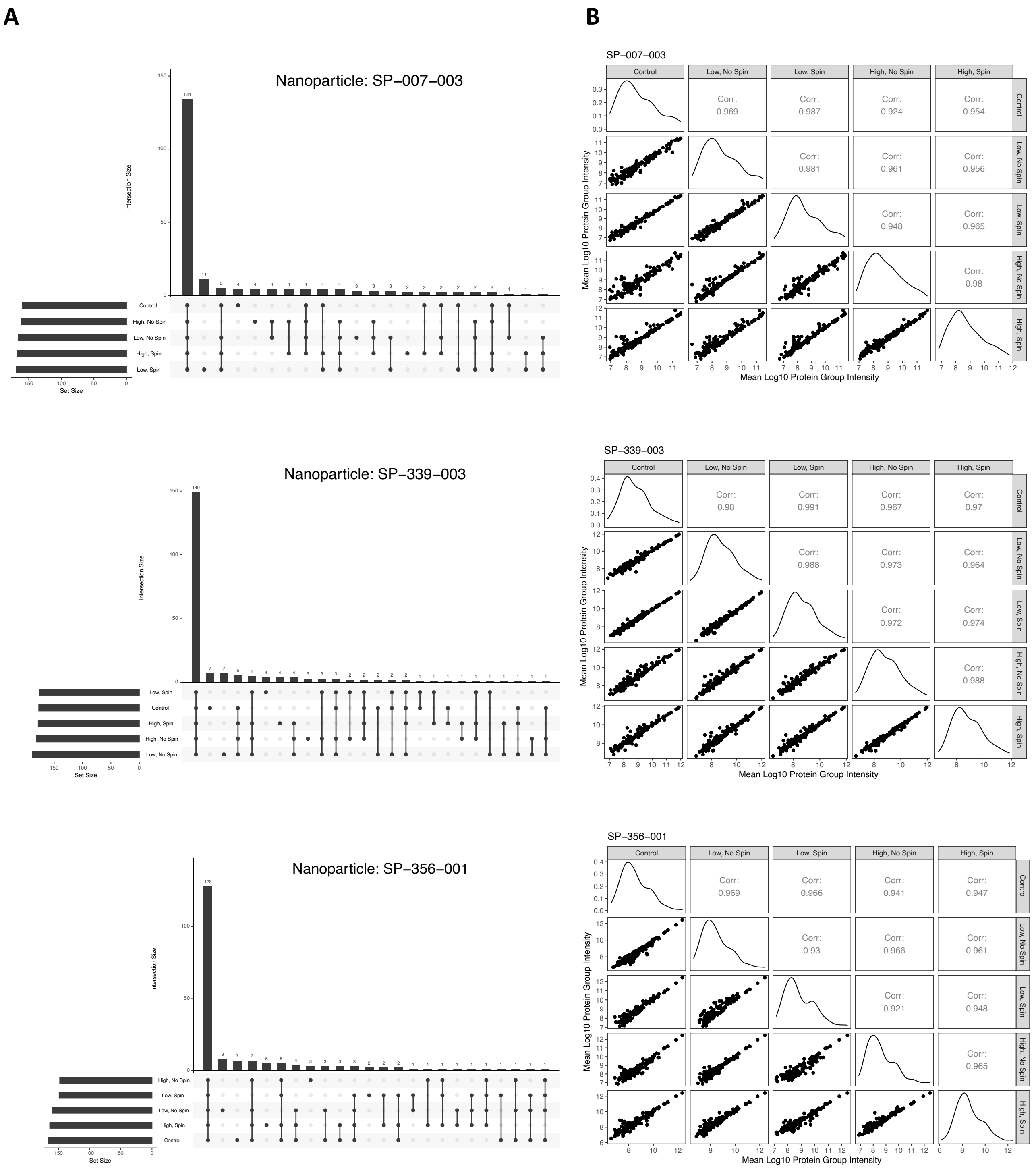

Supplementary Figure 5 Lipid interference

The lipid interference runs tested 2 levels of spiked lipids (low and high), as well as a control with no spiked lipids. Each spike-level/spin condition was run in triplicate and tested against 3

nanoparticles. A) UpSet overlaps were computed for protein groups within each particle across the 5 conditions (Control, Low Spin/No Spin, High Spin/No Spin). The large majority of protein groups were seen in all 5 conditions for all of the particles. B) Scatter plot matrices for the protein group intensities across the three particles are shown below. Good correlation in intensities is seen across the conditions, with correlations typically > 0.95 (slightly lower in SP-356-001). All data was acquired in n = 3 independent assay replicates. Source data are provided as a Source Data file.

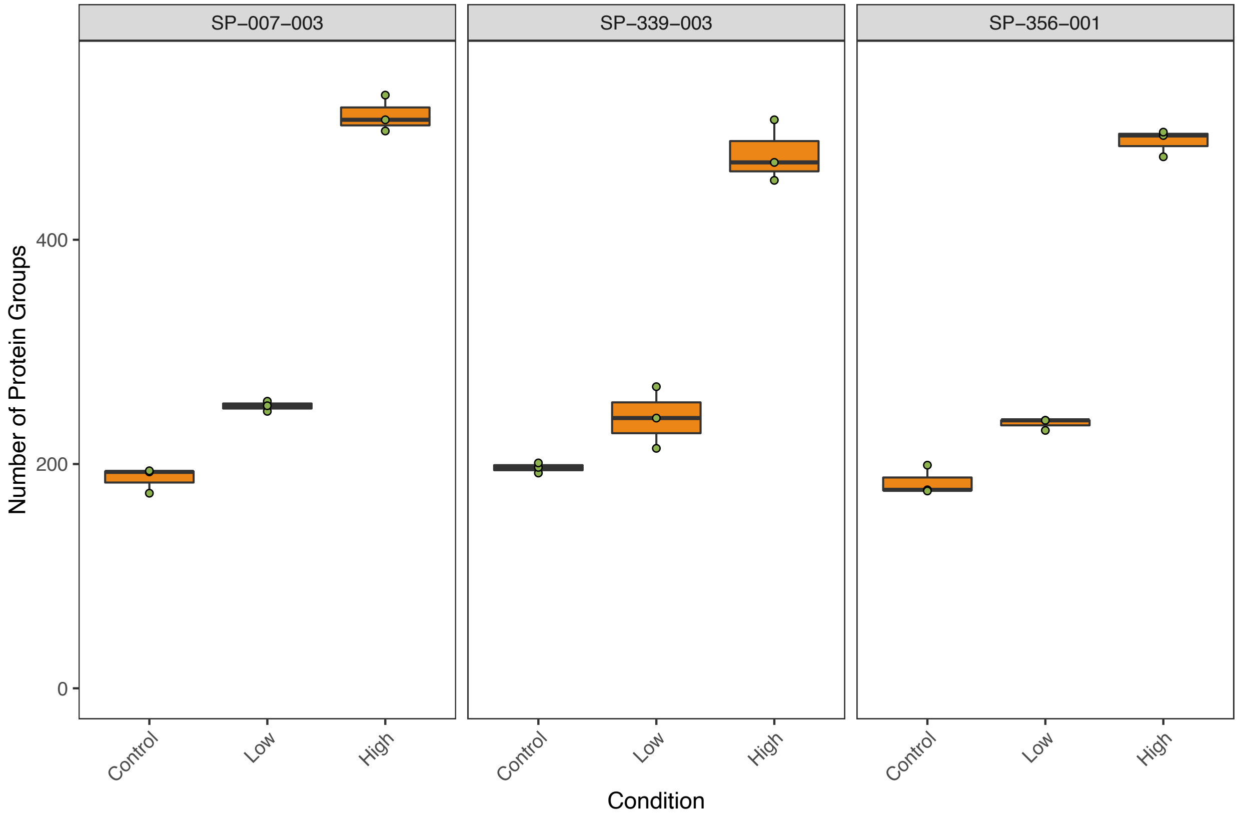

Supplementary Figure 6 Hemolysis interference identification

The hemolysis interference runs tested 2 levels of spiked hemolysate (low and high), as well as a control with no spiked hemolysate (3 different conditions total). Each spike-level condition was run in triplicate and tested against 3 nanoparticles. The number of detected protein groups across the conditions and particles is shown in the figure. There is a clear trend of increasing protein group counts with spike level, which is not unexpected given that additional lysate proteins are being added. Boxplots report the 25% (lower hinge), 50%, and 75% quantiles (upper hinge). Whiskers indicate observations equal to or outside hinge + / – 1.5 * interquartile range (IQR). All data was acquired in n = 3 independent assay replicates. Source data are provided as a Source Data file.

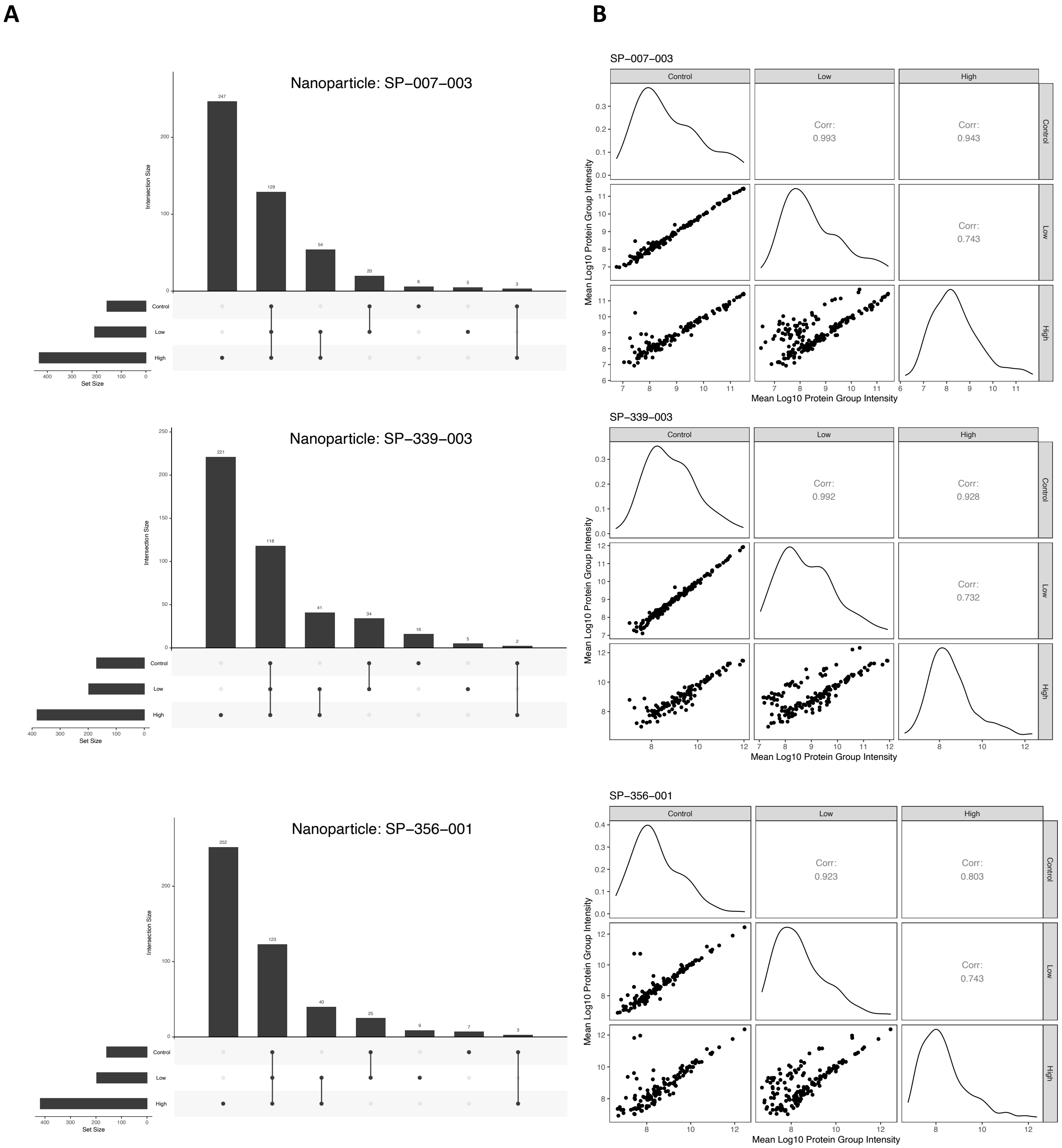

Supplementary Figure 7 Hemolysis interference

A) UpSet overlaps were computed for protein groups within each particle across the 3 conditions. The largest overlap group is for the high spike level, seen in all 3 particles, indicating that the increased protein group counts seen above is largely due to new protein groups not seen in the

other conditions. B) Scatter plot matrices for the protein group intensities across the three particles are shown below. Generally good correlation in intensities is seen across the conditions for the control vs. low spike level conditions, but the correlation decreases in the other comparisons. Interestingly, the correlation between control and high is better than low vs. high. Since the scatter plot pairs only include only common protein groups between the comparisons, it's possible that the control vs. high comparison is missing the additional hemolysate specific proteins and is therefore not showing up on the plot (or contributing to the correlation). In the low vs. high comparison, hemolysate specific proteins can be present in both, and are likely contributing to the group of proteins above the main diagonal (higher in the high spike level) – these are likely the new protein groups appearing with the hemolysate spikes. All data was acquired in n = 3 independent assay replicates. Source data are provided as a Source Data file.

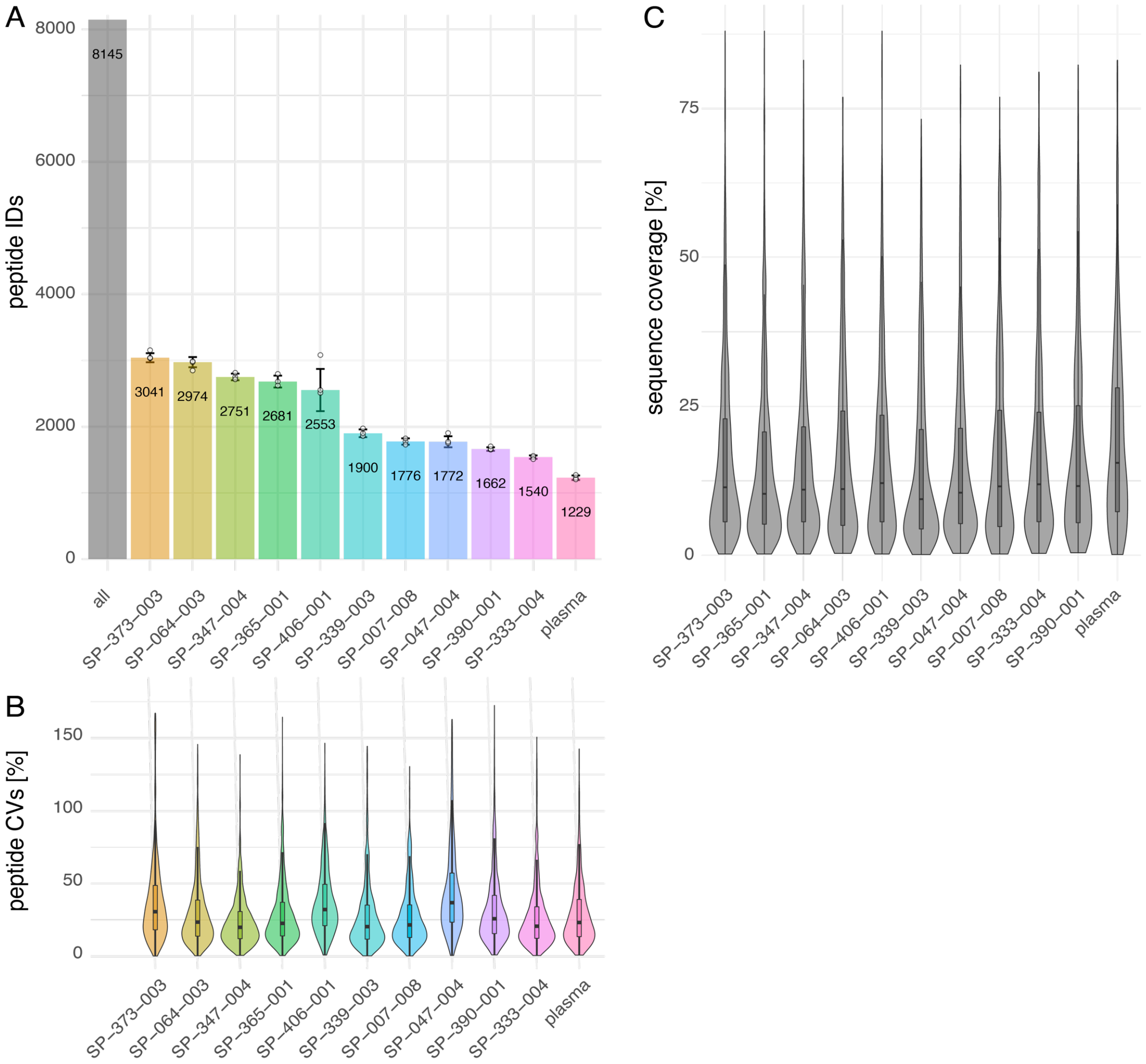

Supplementary Figure 8 Optimized panel of 10 SPIONs in comparison to neat plasma

A) Peptides from the NP corona of 10 SPIONs, quantified by DDA LC-MS/MS (1% peptide FDR). All: number of peptides across all NPs (excluding neat plasma). For respective NPs and plasma, median count and standard deviations across triplicates are shown. White circles show number of protein IDs for each assay replicate. B) CV% distribution (precision) of the NP protein corona-based workflow for neat plasma and 10 SPIONs (filtering for 3 out of 3 valid values across assay replicates). C) Sequence coverage. All inner boxplots report the 25% (lower hinge), 50%, and 75% quantiles (upper hinge). Whiskers indicate observations equal to or outside hinge + / – 1.5 * interquartile range (IQR). Outliers (beyond 1.5 * IQR) are not plotted. Violin plots capture all data points. All data was acquired in n = 3 independent assay replicates. Source data are provided as a Source Data file.

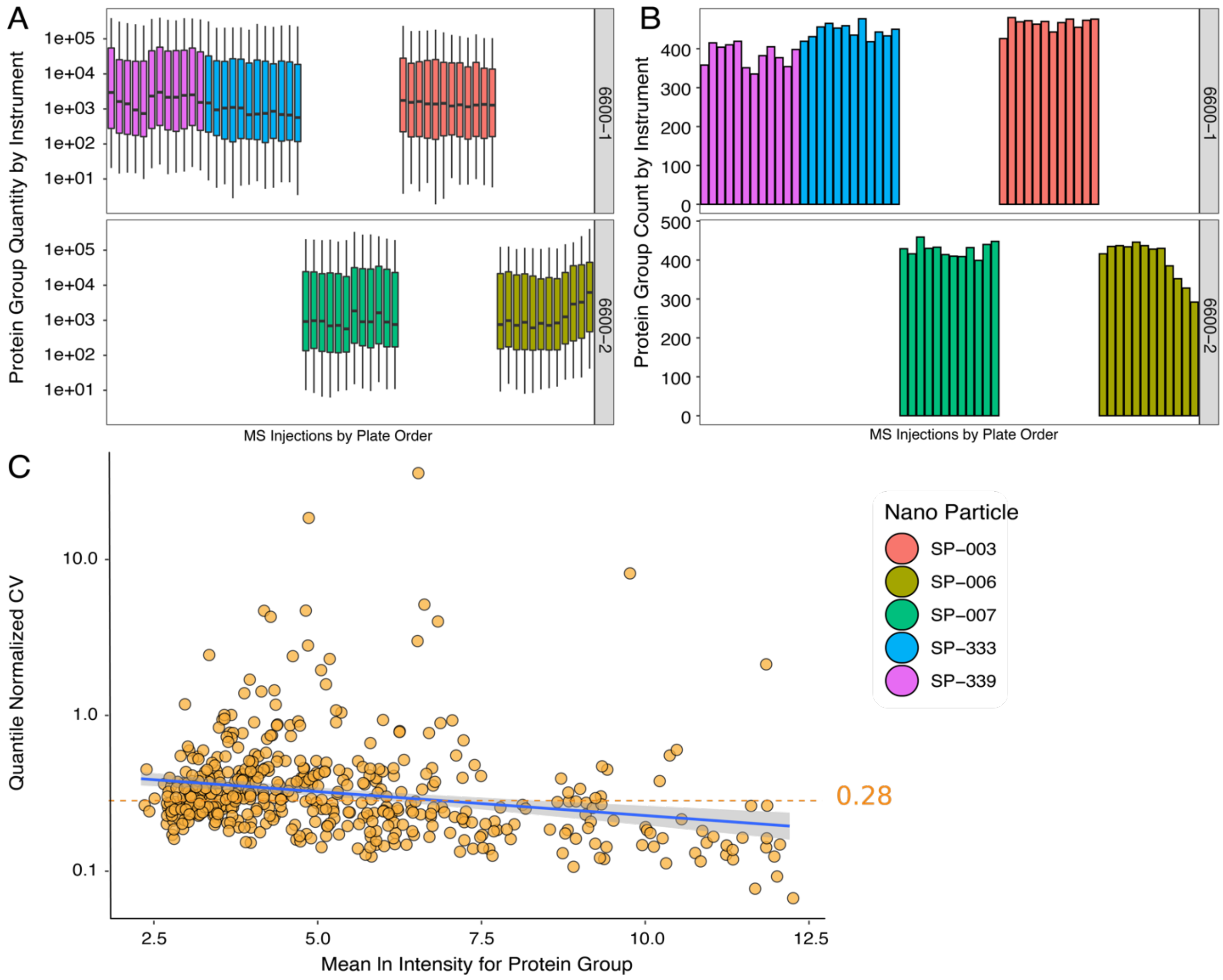

Supplementary Figure 9 QC Sample Characterization

Quality control comparing the same NP interrogating a pooled plasma across more than 1,500 injections on 2 different MS pipelines A) Raw intensity distribution for protein groups quantified. Boxplots report the 25% (lower hinge), 50%, and 75% quantiles (upper hinge). Whiskers indicate observations equal to or outside hinge + / – 1.5 * interquartile range (IQR). B) Number of protein groups at 1% protein and peptide FDR, C) CV of quantile normalized protein intensities (median CV 0.28). Grey shaded regions represent 95% confidence interval of the linear regression model. Source data are provided as a Source Data file.

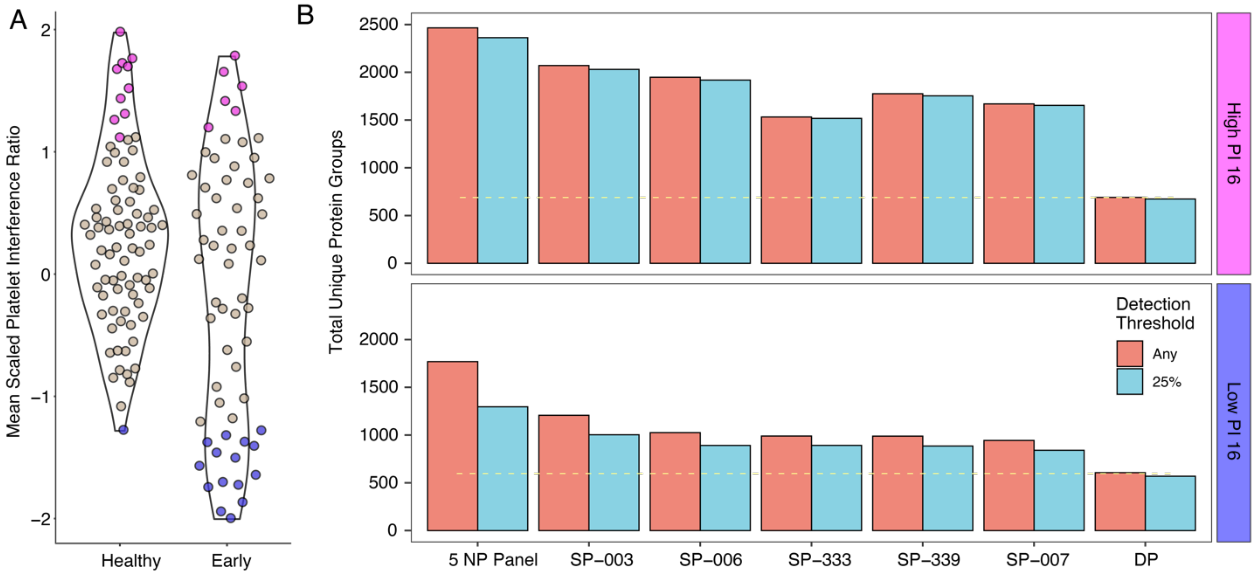

Supplementary Figure 10 5 NP panel performance with respect to platelet index

A) Mean scaled platelet interference ratio (PI) calculated for the 141 subjects. Purple circles indicate the 16 subjects with highest PI, and blue circles the 16 subjects with lowest PI. B) Total number of protein groups in any (red) or more than 25% (blue) of 16 subjects with lowest (lower panel) or highest (upper panel) PI. Yellow dotted line indicates number of IDs in depleted plasma (DP). Source data are provided as a Source Data file.

Supplementary Table 1. Particle size and zeta potential of the three SPIONs: (A) S-003, (B) S-007, and (C) S-011, as measured by dynamic light scattering

**S-003**			
**Measurement #**	**Z-average size**	**PDI**	**Zeta potential**
1	233.8	0.053	-36.4
2	235.3	0.039	-36.8
3	230.4	0.055	-37.4
Average	233 nm	0.05	-37 mV
**S-007**			
**Measurement #**	**Z-average size**	**PDI**	**Zeta potential**
1	284.4	0.049	25.7
2	286.1	0.119	25.9
3	279.7	0.113	25.9
Average	283.4 nm	0.09	+26 mV
**S-011**			
**Measurement #**	**Z-average size**	**PDI**	**Zeta potential**
1	236.5	0.207	0.08
2	238.9	0.198	-0.67
3	237.6	0.201	-0.74
Average	237.7 nm	0.20	0 mV

Supplementary Table 2. Protein group count from the NP corona of the three initial SPIONs, S-003, S-007, and S-011 as determined by DDA LC-MS and MaxQuant (1% protein and peptide FDR). CompleteIDs represent the proteins detected in each of three replicates, "MaxIDs" those detected in any, and "IDs" the median IDs. "All" indicates number of protein groups in any of the replicates across all NPs

**nanoparticles**	**IDs**	**SDs**	**MaxIDs**	**CompleteIDs**
SP-003-001	473	6.244997998	541	410
SP-007-002	432	27.15388247	501	326
SP-011-001	457	14.79864859	529	370
all	755			

Supplementary Table 3. Summary of regression fit of protein intensity as measured by MaxQuant protein group intensity versus measurement by ELISA (See Materials and Methods). Values for individual particles and the average values over the four particles are shown. The proteins are Angiogenin, ANG; C Reactive-Protein, CRP; and Calprotectin, S100A8/9.

Protein	NPs	Intercept	Slope	R^2	Adjusted R^2	Intercept	Slope	R^2	Adjusted R^2	ELISA Endogenous Levels (ug/mL)
ANG	SP-006	0.16	1.05	0.94	0.91	0.3	0.96	1	0.95	0.33
	SP-007	0.75	0.78	0.93	0.9					
	SP-339	0.05	1.05	1	1					
	SP-374	0.23	0.98	0.99	0.99					
CRP	SP-006	-0.24	0.96	1	1	-0.85	1.22	1	0.99	4.9
	SP-007	-0.48	1.07	0.99	0.99					
	SP-339	-1.08	1.31	0.99	0.98					
	SP-374	-1.6	1.54	NA	NA					
S100A8	SP-006	-0.12	1.02	1	1	0.03	0.98	1	0.95	0.89
	SP-007	-0.2	1.12	0.92	0.89					
	SP-339	0.34	0.81	0.99	0.98					
	SP-374	0.1	0.96	0.96	0.95					
S100A9	SP-006	-0.56	1.34	0.9	0.87	-0.09	1.06	0.9	0.92	0.89
	SP-007	-0.44	1.27	0.93	0.91					
	SP-339	0.51	0.68	0.98	0.97					
	SP-374	0.11	0.96	0.95	0.93					

Supplementary Table 4. Median CV% for precision evaluation of the NP protein corona-based workflow for plasma and 10 particles as determined by DDA LC-MS and MaxQuant (1% protein and peptide FDR). Protein group count from the NP corona of the 10 NPs. CompleteIDs represent the proteins detected in each of three replicates, "MaxIDs" those detected in any, and "IDs" the median IDs. "All" indicates number of protein groups in any of the replicates across all NPs

**nanoparticles**	**IDs**	**SDs**	**MaxIDs**	**CompleteIDs**	**Median CV [%]**
plasma	188	8	220	162	*17.1*
SP-007-008	309	7.81024968	365	250	17.1
SP-047-004	352	0.57735027	436	279	30.8
SP-064-003	519	14.7986486	627	433	19.3
SP-333-004	299	10.4403065	344	250	18.2
SP-339-003	383	15.5670592	471	296	17.9
SP-347-004	555	9.45163125	637	469	16.4
SP-365-001	568	22.9419557	677	447	18.4
SP-373-003	604	23.5796522	743	479	25.5
SP-390-001	265	4.72581563	321	216	19.1
SP-406-001	481	73.6545993	653	410	28
all	1189				

Supplementary Table 5. Evaluation of peptides for plasma and 10 particles as determined by DDA LC-MS and MaxQuant (1% peptide FDR). Peptide count from the NP corona of the 10 NPs. CompleteIDs represent the proteins detected in each of three replicates, "MaxIDs" those detected in any, and "IDs" the median IDs. "All" indicates number of protein groups in any of the replicates across all NPs

nanoparticles	IDs	SDs	MaxIDs	CompleteIDs
plasma	1229	33.8575447	1511	976
SP-007-008	1776	47.5008772	2303	1292
SP-047-004	1772	83.2906557	2526	1176
SP-064-003	2974	76.7745618	3786	2112
SP-333-004	1540	25.2388589	1895	1192
SP-339-003	1900	58.7310253	2469	1406
SP-347-004	2751	50.1098793	3445	2105
SP-365-001	2681	89.4725284	3597	1867
SP-373-003	3041	68.2446579	4214	2091
SP-390-001	1662	27.0246801	2106	1265
SP-406-001	2553	318.236285	3588	1947
all	8145			

Supplementary Table 6. Number of FDA cleared biomarkers matching to 10 SPIONs and neat plasma

nanoparticle	# Biomarkers
SP-007-008	38
SP-339-003	33
plasma	40
SP-373-003	43
SP-390-001	40
SP-333-004	41
SP-347-004	38
SP-365-001	36
SP-406-001	42
SP-047-004	43
SP-064-003	41
All NPs	53

Supplementary Table 7. 5 NP panel protein identification performance in 141 subjects

nanoparticle	total	mean	sd	total_25
Panel	2499	1663.51064	348.456446	1992
SP-003	2101	1237.91489	351.589076	1633
SP-006	1969	1081.83688	372.249084	1476
SP-339	1803	897.390071	288.673661	1170
SP-007	1698	896.943262	275.62702	1193
SP-333	1546	738.212766	186.788414	920
Depleted Plasma	740	418.87234	49.4921135	503

Supplementary Table 8. 5 NP panel performance with respect to platelet index (PI). ID threshold indicates the level of completeness for identifying a protein across all subjects.

nanoparticle	total	ID threshold	mean	sd	pi_level
Panel	1769	Any	1020.1875	82.7399289	Low PI 16
DP	606	Any	373.875	37.6738194	Low PI 16
SP-003	1207	Any	608.875	87.794362	Low PI 16
SP-006	1025	Any	486.75	99.195094	Low PI 16
SP-007	944	Any	466.75	38.976061	Low PI 16
SP-333	990	Any	545.1875	50.6336104	Low PI 16
SP-339	989	Any	515.125	55.4254755	Low PI 16
Panel	1296	25%	971.875	68.2083817	Low PI 16
DP	569	25%	370.25	36.1303197	Low PI 16
SP-003	1003	25%	592.0625	78.3985278	Low PI 16
SP-006	891	25%	476.5	93.2837964	Low PI 16
SP-007	841	25%	459	35.7211422	Low PI 16
SP-333	892	25%	536.6875	47.7182268	Low PI 16
SP-339	885	25%	506.625	52.1585723	Low PI 16
Panel	2465	Any	2127.75	67.3027984	High PI 16
DP	690	Any	492.625	55.5144125	High PI 16
SP-003	2070	Any	1692.4375	112.747487	High PI 16
SP-006	1948	Any	1592.9375	82.5198713	High PI 16
SP-007	1669	Any	1334.5625	103.786299	High PI 16
SP-333	1532	Any	1069.875	235.550384	High PI 16
SP-339	1775	Any	1401.4375	134.453446	High PI 16
Panel	2362	25%	2115	66.3556077	High PI 16
DP	673	25%	490.5625	54.5881168	High PI 16
SP-003	2030	25%	1687.8125	112.404756	High PI 16
SP-006	1918	25%	1589.5	81.6284264	High PI 16
SP-007	1654	25%	1333.1875	103.828846	High PI 16
SP-333	1517	25%	1068.375	235.207674	High PI 16
SP-339	1753	25%	1398.8125	134.516526	High PI 16

SEERPRO: A Prospective Blood Sample Collection Study to Evaluate a Panel of Protein-based Biomarkers

Subject enrollment and sample collection

- Subjects may be enrolled and samples collected Monday through Thursday; there is no Friday enrollment (contact Seer for questions about holiday scheduling)
- Streck tubes are shipped on the day of collection or if collected late in the day, the next day
- Samples should only be shipped Monday through Thursday

Subject and sample numbering conventions

Separate each set of numbers by hyphen:

Site number	Subject ID	Tube number	Aliquot
001, 002…	0001, 0002…	EDTA: 1, 2<br>SST: 3<br>PAXgene: 4<br>Streck: 5	Plasma: 01-10<br>Buffy coat: 99<br>Serum: 01-10
Example: site 001, first patient<br>Subject ID: 001-0001<br>k2 EDTA tube 1: 001-0001-1<br>k2 EDTA tube 1 plasma aliquots: 001-0001-1-01, 001-0001-1-02…<br>k2 EDTA tube 1 buffy coat aliquot: 001-0001-1-99<br>k2 EDTA tube 2: 001-0001-2<br>k2 EDTA tube 2 plasma aliquots: 001-0001-2-01, 001-0001-2-02…<br>k2 EDTA tube 2 buffy coat aliquot: 001-0001-2-99<br>SST tube 3: 001-0001-3<br>Serum aliquots: 001-0001-3-01, 001-0001-3-02…<br>PAXgene tube 4: 001-0001-4 (no aliquot IDs)<br>Streck tube 5: 001-0001-5 (no aliquot IDs)			

Supplies

1. Venipuncture equipment: 21g butterfly needle, vacutainer holder, alcohol prep pad, tourniquet, 2x2 sterile gauze, bandage
2. Cryolabels
3. Blood tubes (5)
   a) 2 k2 EDTA
   b) 1 serum separator (SST)
   c) 1 PAXgene
   d) 1 Streck cell-free DNA BCT
4. 8 5.8mL pipettes (2 mL volume with 500 uL graduation)
5. 3 15mL conical Falcon tubes
6. 30 2.0mL cryovials
7. 9x9 cryobox
8. Ice for transporting k2 EDTA tubes until centrifuge and between aliquot and freeze
9. Label tubes using provided labels and numbering conventions
   a) Label all collection tubes, including EDTA and SST
   b) Label all aliquot tubes
   c) You do not need to label the Falcon tubes

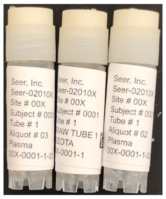

K2 EDTA

1. Immediately after tubes are full, slowly invert 8-10 times
2. Centrifuge within 30 minutes of collection at 1100-1300g for 15 minutes
3. Immediately following centrifugation, place tubes on ice until samples are aliquoted
4. Using a 5.8 mL pipette, remove the plasma from each k2EDTA tube using care to not disturb the buffy coat layer
5. Transfer the plasma to a 15 mL conical Falcon tube; invert the Falcon tube 5 times to mix, then put the 15 mL Falcon tube on ice

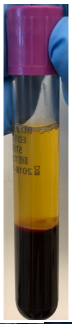

6. Using a second 5.8 mL transfer pipette, transfer 500 uL aliquots into labelled 2.0 mL cryovials
   a) Draw up 1 mL into the transfer pipette (up to the bulb)
   b) Dispense 2 500 uL aliquots into 2 cryovials
   c) Repeat, making as many 500 uL aliquots as possible; you will have about 8-10 aliquots
   d) If left with less than 500 uL after aliquoting, fill the last cryovial tube with whatever volume is remaining
   e) Keep aliquots on ice until they are transferred to freezer

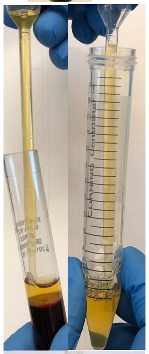

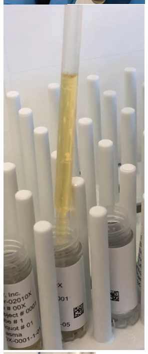

7. Using a third 5.8 mL transfer pipette, remove the buffy coat layer and transfer to a 2.0 mL cryovial
8. Repeat the process for the second k2EDTA tube
9. Immediately following aliquoting, place the cryovial tubes in ice until transfer to the –20°C / –70°C / –80°C freezer
10. Within 1 hour of completion of centrifugation, transfer cryovial tubes to a 9x9 cardboard cryobox, and place cryobox in the –20°C / –70°C / –80°C freezer
11. k2 EDTA tubes may be discarded

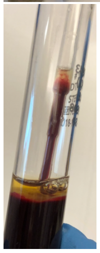

Serum separator (SST)

1. Allow blood to clot for at least 30 min but not more than 1 hour at room temperature
2. Centrifuge samples at 1100-1300g for 20 minutes
3. Using a 5.8 mL pipette, remove the serum from the SST tube and transfer to a 15 mL conical Falcon tube; invert the Falcon tube 5 times to mix
4. Using a second 5.8 mL transfer pipette, transfer 500 uL aliquots to pre-labelled 2.0 mL labelled cryovials
   a) Draw up 1 mL into the transfer pipette (up to the bulb)
   b) Dispense 2 500 uL aliquots into 2 cryovials
   c) Repeat, making as many 500 uL aliquots as possible; you will have about 8-10 aliquots
   d) If left with less than 500 uL after aliquoting, fill the last cryovial tube with whatever volume is remaining
5. Within 1 hour of completion of centrifugation, transfer cryovial tubes to a 9x9 cardboard cryobox, and place cryobox in the –20°C / –70°C / –80°C freezer
6. SST tube may be discarded

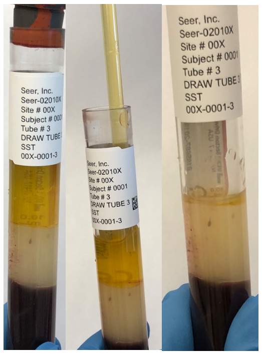

PAXgene

1. Allow at least 10 seconds for a complete tube draw, and ensure that the blood has stopped flowing before stopping blood draw
2. Immediately after collection, gently invert the tube 8-10 times
3. Store upright at room temperature (18-25°C) for 2-72 hours
4. Transfer to –20°C / –70°C / –80°C freezer; store in a wire (not Styrofoam) rack

Cell-free DNA Streck BCT

1. Fill tube completely
2. Immediately after collection, gently invert the tube 8-10 times
3. Store upright at room temperature until packaged for shipment; tubes must not be refrigerated or frozen
4. Ship on the day of collection or, if collected late in the day, the next day

Recordkeeping: record the following on the Sample Requisition Form

1. Subject ID
2. Date collected
3. Time collected
4. K2 EDTA tubes
   a) Time centrifuge started
   b) Time centrifuge complete
   c) Time aliquots placed in –80°C freezer
5. SST tube
   a) Time centrifuge started
   b) Time centrifuge complete
   c) Time aliquots placed in –80°C freezer
6. PAXgene tube: date and time placed in –80°C freezer
7. Send a copy of the Sample Requisition Form to Lyssa Friedman; retain the original in the study binder

Shipping

1. Ship samples using shipping materials provided
2. Ship samples on Monday through Thursday only; special arrangements will be made for holiday shipping schedules
3. Store samples as described above until they are packaged for shipment
4. Streck tubes are shipped on the day of collection at ambient temperature in ambient shipper
5. Plasma, serum and buffy coat cryovials, PAXgene: are shipped at least monthly on dry ice in frozen shipper
6. Assign a consecutive shipping number: 1, 2, 3…
7. Complete the following on the Sample Requisition Form:
   a) Date of shipment
   b) Time samples packaged for shipment
   c) FedEx tracking number
   d) All aliquots and applicable tubes included in shipment (Yes/No); if discrepancy (e.g., missing tube), list missing ID number or applicable discrepancy
8. Send a copy of the Sample Requisition Form to Lyssa Friedman
9. Include a copy of the Sample Requisition Form in the shipment – pack between the inner Styrofoam and outer layers
10. Samples are shipped priority overnight to:

William Manning, PhD
Seer
170 Harbor Way
South San Francisco, CA 94080
Phone: (650) 453-0903
Email: / bmanning@seer.bio

Frozen shipping (plasma, buffy coat and serum cryovials, PAXgene tubes)

1. Prepare a frozen shipping box with at least 10-15 pounds of dry ice
2. Place cryovial and PAXgene tubes in Ziploc bag containing absorbent sheet, ensuring that dry ice is packed around the sample tubes
3. Place a copy of the Sample Requisition Form(s) between the inner cooler and outer box prior to sealing

Ambient shipping (Streck tube)

1. Ship on the day of collection or, if collected late in the day, the next day
2. Use an ambient shipping box; samples may be batch shipped
3. Place Streck tubes in Ziploc bag containing absorbent sheet
4. Place a copy of the Sample Requisition Form(s) between the inner cooler and outer box prior to sealing

Supplementary Note 2. 2020 Competing Interests Disclosure of Dr. Robert Langer

From FY 2015 to the present, Dr. Robert Langer receives licensing fees (to patents in which he was an inventor on) from, invested in, consults (or was on Scientific Advisory Boards or Boards of Directors) for, lectured (and received a fee), or conducts sponsored research at MIT for which he was not paid for the following entities:

1. 7th Sense;
2. Abpro Labs;
3. Abpro-Korea;
4. Acorda (Formerly Civitas Therapeutics);
5. Aleph Farms;
6. Alivio Therapeutics;
7. Alkermes;
8. Allevi;
9. Alnylam Pharmaceuticals, Inc;
10. Apotex;
11. Arcadia Biosciences, Inc;
12. Arsenal Medical;
13. Artificial Cell Technology, Inc;
14. Avalon-Globocare;
15. BASF Corporation;
16. Biogen;
17. BioInnovation Institute (Novo Nordisk Founden);
18. Blackstone (Formerly Clarus);
19. Boston Children's Hospital;
20. Celero;
21. Cellomics Technology, Llc;
22. Cellular Biomedical;
23. Charles River Laboratories, Inc.;
24. Clontech Laboratories;
25. Combined Therapeutics ("CTx");
26. Conference Forum;
27. Cornell University;
28. Crispr Therapeutics Ag;
29. Crown Bioscience Inc.;
30. Cygnal Therapeutics, Inc.;
31. Daros, Inc.
32. Dare Biosciences (Formerly Microchips Biotech, Juniper Pharmaceuticals, and Columbia Laboratories);
33. DeepBiome;
34. Dewpoint Therapeutics;
35. Dispendix/ Cellink;
36. Domain;
37. Eagle Pharmaceuticals;
38. Edigene Biotechnology, Inc.;
39. Editas Medicine, Inc.;
40. Eli Lilly;
41. Eisai Inc.;
42. Entrega;
43. Everlywell;
44. Evox Therapeutics, Ltd.;
45. Flagship Pioneering;
46. Frequency Therapeutics, Inc.;
47. Genemedicine Co Lmtd;
48. GenScript USA Inc;
49. GENUV;
50. Glaxosmithkline Llc;
51. Glycobia;
52. Glympse Bio;
53. Greenlight Biosciences;
54. HCR (HealthCare Royalty Partners);
55. Hopewell Therapeutics;
56. Horizon Discovery Group Plc;
57. Humacyte, Inc.;
58. IBEX Pharmaceuticals, Inc.;
59. ImmuneXcite Inc.;
60. Indivor;
61. Institute of Immunology Co. Ltd;
62. Integrated Dna Technologies, Inc.;
63. InVivo Therapeutics;
64. IxBio;
65. J.R. Simplot Company;

From FY 2015 to the present, Dr. Robert Langer receives licensing fees (to patents in which he was an inventor on) from, invested in, consults (or was on Scientific Advisory Boards or Boards of Directors) for, lectured (and received a fee), or conducts sponsored research at MIT for which he was not paid for the following entities:

66. Jnana Therapeutics;
67. Kala Pharmaceuticals;
68. Kallyope, Inc.;
69. Kensa;
70. Kodikaz Therapeutics
71. Ksq Therapeutics, Inc.;
72. Landsdowne Labs;
73. LikeMinds;
74. Luminopia, Inc.;
75. Luye (Shandong luye);
76. Lyndra Therapeutics;
77. Lyra Therapeutics (Formerly "480 Biomedical");
78. McGovern Institute;
79. Medikinetics Co., Ltd.;
80. Merck;
81. MGH Ragon Institute;
82. Micelle;
83. Moderna Therapeutics;
84. Momenta;
85. Monsanto Company;
86. Muse Biotechnologies Inc.
87. Mylan;
88. Nanobiosym;
89. Nanobiotix;
90. Newbridge Ventures LLC;
91. Noveome Biotherapeutics, Inc.;
92. Novo Nordisk;
93. Particles for Humanity;
94. Pfizer, Inc.;
95. Pioneer Hi-Bred International, Inc.;
96. Polaris Partners;
97. Portal Instruments;
98. Preceres, Llc (Acquired by Monsanto);
99. Pulmatrix;
100. PureTech;
101. ReLive;
102. Reprocell Usa, Inc. (Formerly Stemgent);
103. Rubius Therapeutics;
104. Secant Medical, Inc.;
105. Seer, Inc.;
106. Selecta Biosciences;
107. Senses LLC;
108. Setsuro Tech Inc.;
109. Seventh Sense Biosystems, Inc.;
110. Shenzhen Rice Life Technology, Ltd;
111. Shire Ag;
112. Siglion;
113. Sigma Aldrich Co. Llc;
114. Sio2;
115. Ske S.R.L.;
116. Soil Culture Solutions Llc (Dba Soilcea);
117. SQZ Biotechnologies;
118. StemBioSys, Inc.;
119. SuonoBio;
120. T2 Biosystems;
121. Taconic Biosciences, Inc. (formerly Taconic Farms);
122. TARA;
123. Tarveda Therapeutics;
124. Tesio Pharmaceuticals
125. Third Rock;
126. Tiba Biotech Llc;
127. TissiuM (formerly"Gecko");
128. Transgenic Inc.;
129. Translate Bio (Formerly Rana Therapeutics, Inc.);
130. Trilink Biotechnologies, Inc.;
131. Unilever (Living Proof);

From FY 2015 to the present, Dr. Robert Langer receives licensing fees (to patents in which he was an inventor on) from, invested in, consults (or was on Scientific Advisory Boards or Boards of Directors) for, lectured (and received a fee), or conducts sponsored research at MIT for which he was not paid for the following entities:

132. VasoRX;
133. Verseau Therapeutics, Inc.;
134. Vivtex Corporation;
135. Whitehead Institute;
136. Wiki Foods;
137. Yz Biosciences (Guangzhou) Inc.;
138. Zenomics;
