# Supplementary material for: Rapid, deep and precise profiling of the plasma proteome with multi-nanoparticle protein corona
Source: Nat Commun. 2020 Jul 22;11:3662. doi: 10.1038/s41467-020-17033-7 (PMC7376165; doi:10.1038/s41467-020-17033-7)
Supplement: Supplementary file 2 — Description of Additional Supplementary Files [file 41467_2020_17033_MOESM2_ESM.docx]

**Description of Additional Supplementary Files**

**File Name: Supplementary Data 1**

**Description:** 3 NP protein groups from MaxQuant

**File Name: Supplementary Data 2**

**Description:** Spike-in Experiments protein groups from MaxQuant

**File Name: Supplementary Data 3**

**Description:** Characterization of 43 nanoparticle library used for particle screen

**File Name: Supplementary Data 4**

**Description:** 10 NP protein groups from MaxQuant

**File Name: Supplementary Data 5**

**Description:** NSCLC study protein groups Spectronaut

**File Name: Supplementary Data 6**

**Description:** Sample identifier and annotations

**File Name: Supplementary Data 7**

**Description:** IRBs

**File Name: Supplementary Data 8**

**Description:** NSCLC sample annotations
