# Supplementary material for: Rapid, deep and precise profiling of the plasma proteome with multi-nanoparticle protein corona
Source: Nat Commun. 2020 Jul 22;11:3662. doi: 10.1038/s41467-020-17033-7 (PMC7376165; doi:10.1038/s41467-020-17033-7)
Supplement: Supplementary file 11 — Reporting Summary [file 41467_2020_17033_MOESM11_ESM.pdf]

nature research

Corresponding author(s): John E. Blume
Vivek Farias
Omid C. Farokhzad

Last updated by author(s): Apr 18, 2020

Reporting Summary

Nature Research wishes to improve the reproducibility of the work that we publish. This form provides structure for consistency and transparency in reporting. For further information on Nature Research policies, see Authors & Referees and the Editorial Policy Checklist .

Statistics

For all statistical analyses, confirm that the following items are present in the figure legend, table legend, main text, or Methods section.

n/a	Confirmed	
☐	☒	The exact sample size (*n*) for each experimental group/condition, given as a discrete number and unit of measurement
☐	☒	A statement on whether measurements were taken from distinct samples or whether the same sample was measured repeatedly
☐	☒	The statistical test(s) used AND whether they are one- or two-sided<br>*Only common tests should be described solely by name; describe more complex techniques in the Methods section.*
☒	☐	A description of all covariates tested
☐	☒	A description of any assumptions or corrections, such as tests of normality and adjustment for multiple comparisons
☐	☒	A full description of the statistical parameters including central tendency (e.g. means) or other basic estimates (e.g. regression coefficient) AND variation (e.g. standard deviation) or associated estimates of uncertainty (e.g. confidence intervals)
☒	☐	For null hypothesis testing, the test statistic (e.g. *F*, *t*, *r*) with confidence intervals, effect sizes, degrees of freedom and *P* value noted<br>*Give P values as exact values whenever suitable.*
☒	☐	For Bayesian analysis, information on the choice of priors and Markov chain Monte Carlo settings
☒	☐	For hierarchical and complex designs, identification of the appropriate level for tests and full reporting of outcomes
☒	☐	Estimates of effect sizes (e.g. Cohen's *d*, Pearson's *r*), indicating how they were calculated

Our web collection on statistics for biologists contains articles on many of the points above.

Software and code

Policy information about availability of computer code

Data collection	Mass spectrometry (MS) instrument-control software was provided by the instrument vendor (i.e., Thermo Fisher XCalibur)
Data analysis	Primary MS DDA data analysis was performed with MaxQuant/Andromeda (v1.6.7), DIA data was processed with Spectronaut ((v13.8.190930.43655), R3.6) as described and cited in the manuscript. Routine statistical analysis and data visualization was performed in R as cited and data were visualized using the ggplot2 package (H. Wickham. ggplot2: Elegant Graphics for Data Analysis. Springer-Verlag New York, 2016.)

For manuscripts utilizing custom algorithms or software that are central to the research but not yet described in published literature, software must be made available to editors/reviewers. We strongly encourage code deposition in a community repository (e.g. GitHub). See the Nature Research guidelines for submitting code & software for further information.

Data

Policy information about availability of data

All manuscripts must include a data availability statement. This statement should provide the following information, where applicable:

- Accession codes, unique identifiers, or web links for publicly available datasets
- A list of figures that have associated raw data
- A description of any restrictions on data availability

The mass spectrometry proteomics data have been deposited to the ProteomeXchange Consortium via the PRIDE partner repository with the dataset identifier PXD017052 and will be available for download upon manuscript publication.

Field-specific reporting

Please select the one below that is the best fit for your research. If you are not sure, read the appropriate sections before making your selection.

- [x] Life sciences
- [ ] Behavioural & social sciences
- [ ] Ecological, evolutionary & environmental sciences

For a reference copy of the document with all sections, see nature.com/documents/nr-reporting-summary-flat.pdf

Life sciences study design

All studies must disclose on these points even when the disclosure is negative.

Sample size	No group comparisons were made in order to determined a specified effect.
Data exclusions	No data were excluded from the methods analysis, all analyses were performed as described in the Materials and Methods.
Replication	No comparative result or between-group comparison was determined, so no separate replication or validation of a discovered, comparative result is appropriate.
Randomization	In this method development and descriptive study no sample group univariates are relevant for the analysis.
Blinding	In this method development and descriptive study no blinding of the data was applied or appropriate as no novel comparative group effects were determined.

Reporting for specific materials, systems and methods

We require information from authors about some types of materials, experimental systems and methods used in many studies. Here, indicate whether each material, system or method listed is relevant to your study. If you are not sure if a list item applies to your research, read the appropriate section before selecting a response.

Materials & experimental systems

n/a	Involved in the study	
x		Antibodies
x		Eukaryotic cell lines
x		Palaeontology
x		Animals and other organisms
	x	Human research participants
	x	Clinical data

Methods

n/a	Involved in the study	
x		ChIP-seq
x		Flow cytometry
x		MRI-based neuroimaging

Human research participants

Policy information about studies involving human research participants

Population characteristics	Study population in all groups included men and women ≥ 18 years of age who do not have any prior history of any type of cancer and willing to consent to provide blood via venipuncture. The patients also should not also be taking any biological therapies. Additionally, for the NSCLC group of patients, they require a pathology confirmed diagnosis and they should have no prior therapy for the newly diagnosed cancer, including surgery, radiotherapy, chemotherapy, immunotherapy, targeted therapy, hormonal therapy, or other experimental therapy. For the pulmonary co-morbid control group patients, additional criteria included that they do not have any current cancer but have one or more of the following conditions such as COPD, Emphysema, Pulmonary fibrosis, Asthma and any other chronic lung disease. The subjects enrolled in the healthy control group have no documentation of any of the clinical diagnoses of cancer or findings in the pulmonary co-morbidities
Recruitment	All subjects are screened for inclusion/exclusion criteria and subjects will be enrolled sequentially into the enrollment groups.
Ethics oversight	The study is conducted at 24 sites and the study was approved for 21 sites by WIRB. 3 sites received approval from their local institutional IRB which are Vanderbilt Institutional Review Board, Meritus Medical Center Institutional Review Board and Baylor Scott&White Research IRB

Note that full information on the approval of the study protocol must also be provided in the manuscript.

Clinical data

Policy information about clinical studies

All manuscripts should comply with the ICMJE guidelines for publication of clinical research and a completed CONSORT checklist must be included with all submissions.

Clinical trial registration	The study is currently not registered in clinicaltrials.gov as this is an observational study and not an applicable study that requires registration in Clincaltrials.gov. The study is conducted under IRB approved protocols
Study protocol	SEERPRO: A Prospective Blood Sample Collection Study to Evaluate a Panel of Protein-based Biomarkers - (Sample collection protocol made available as supplemental information)
Data collection	The locales of data collection and the recruitment period for each site is provided in the table provided. The data collection began on 11/14/18 and data collection completion was on 10/02/19
Outcomes	This is not an interventional trial and hence there are no outcomes.
